# Mycobacteria modulate SUMOylation to suppresses protective responses in dendritic cells

**DOI:** 10.1371/journal.pone.0283448

**Published:** 2023-09-29

**Authors:** Vandana Anang, Aayushi Singh, Ankush Kumar Rana, Shakuntala Surender Kumar Saraswati, Upasana Bandyopadhyay, Chaitenya Verma, Attinder Chadha, Krishnamurthy Natarajan

**Affiliations:** Infectious Disease Immunology Lab, Dr. B.R. Ambedkar Center for Biomedical Research, University of Delhi, Delhi, India; Centenary Institute, AUSTRALIA

## Abstract

Post translational modifications (PTMs) are exploited by various pathogens in order to escape host immune responses. SUMOylation is one of the PTMs which is involved in regulation of a variety of cellular responses. However, the effects of host SUMOylation on pathogenic bacteria largely remain elusive. We, therefore, investigated the role of SUMOylation in regulating defense responses in dendritic cells (DCs) during mycobacterial infection. Dendritic Cells of female BALB/c mice and THP-1 macrophages were used. Western blotting was performed to measure the expression of level of SUMO1, pSTAT1, pp38, pERK, Beclin-1, LC3, Bax and Cytochrome C. For bacterial burden confocal microscopy and CFU (Colony Forming Unit) were used. Flow cytometry was used for ROS and co-stimulatory molecules measurement. Cytokine level were measured using ELISA. We show that stimulation of Bone Marrow Derived Dendritic Cells (BMDCs) with mycobacterial antigen Rv3416 or live infection with *Mycobacterium bovis* BCG increases the SUMOylation of host proteins. Inhibition of SUMOylation significantly decreased intracellular bacterial loads in DCs. Additionally, inhibiting SUMOylation, induces protective immune responses by increasing oxidative burst, pro-inflammatory cytokine expression and surface expression of T cell co-stimulatory molecules, and activation of pSTAT1 and Mitogen Activated Protein Kinases (MAPK) proteins- pp38 and pERK. SUMOylation inhibition also increased apoptosis and autophagy in BMDCs. Intriguingly, mycobacteria increased SUMOylation of many of the above molecules. Furthermore, inhibiting SUMOylation in DCs primed T cells that in turn attenuated bacterial burden in infected macrophages. These findings demonstrate that SUMOylation pathway is exploited by mycobacteria to thwart protective host immune responses.

## Introduction

*Mycobacterium tuberculosis* (*M*. *tb*) continues to cause morbidity and mortality, killing 1.5 to 2.0 million people annually, and latently infect an estimated one quarter of the world’s population [1]. Although *M*. *tb* resides mainly within the macrophages, studies have shown that DCs can also phagocytose the bacterium and are crucial to initiate protective immune responses affecting mycobacterial survival in the host [2–5]. We previously identified several genes in DCs that play critical roles in modulating defense functions during *M*. *tb* infection [6]. One of the genes that played a major role in regulating defense functions is *Senp8*. SENP8 is a cysteine protease involved in neddylation, a post-translational modification pathway [7, 8]. We recently reported the role of neddylation during *M*. *tb* infection [9]. Stimulation with *M*. *tb* proteins increased neddylation in DCs. Knockdown of specific genes of the neddylation pathway resulted in induction of protective responses like oxidative burst, phagosome-lysosome fusion, apoptosis and autophagy [9].

SUMOylation is a post-translational modification that involves the covalent addition of a ubiquitin-like protein called SUMO (Small Ubiquitin-like Modifier) on specific targets. Similar to ubiquitination and neddylation, it is composed of an E1 SUMO enzyme (SAE1/2 heterodimer), an E2 SUMO enzyme (Ubc9) and E3 SUMO enzyme. DeSUMOylases and SUMO-specific proteases tightly regulate the SUMOylation levels of specific target proteins [7]. Many reports show the involvement of SUMOylation pathway in *Salmonella typhimurium*, *Listeria monocytogenes*, *Clostridium perfringens* and *Streptococcus pneumoniae* infections [10–12]. Specifically, *Salmonella* infection induced SUMOylation of RAB7. Blocking RAB7 SUMOylation altered *Salmonella* pathogenesis [10] indicating that pathogens subvert SUMOylation of host proteins to its advantage. On the contrary, *Listeria monocytogenes* infection results in a decreased SUMOylation in cells by mediating proteosome independent degradation of Ubc9, a critical protein in the SUMOylation pathway [11]. These reports clearly show that pathogens modulate the SUMOylation pathway in cells to their advantage.

Keeping the above in mind, in this study, we show that stimulation of BMDCs with Rv3416 or infection with *M*. *bovis* BCG increases the SUMOylation of host proteins. Inhibiting SUMOylation pathway reduces bacterial burden by increasing the activation of proteins involves in cell signaling like pERK, pp38 and pSTAT1, oxidative burst, expression of co-stimulatory molecules and pro-inflammatory cytokines and promotion of apoptosis and autophagy. Finally, inhibiting SUMOylation in DCs, activated T cells, that in turn activated mycobacteria infected macrophages to reduce bacterial survival. Collectively, our data point towards a negative role of SUMOylation in mediating protective responses to mycobacterial infection.

## Material and methods

### Animals

Female BALB/c mice of 4–6 week of age were kept in a pathogen-free environment. All experiments were conducted following approval from the institutional animal ethics committee of Dr. B. R. Ambedkar Center for Biomedical Research, University of Delhi, and in compliance with the national and ARRIVE guidelines. The procedures used in the study were carried out by well-experienced competent workers trained to perform them. All efforts were made to minimize suffering and pain.

### Materials

Fluorescence-tagged antibodies against mouse/human CD40 (PE) and PDL-1 (PE/FITC) were obtained from BD Biosciences. Antibodies to SUMO1, pp38, pERK, pSTAT1, Bax, Cytochrome C, β-actin, GAPDH and siRNAs to *Sumo1* and *Ubc9* were from Santa Cruz Biotechnologies (Santa Cruz, CA). Antibodies to p38, pp38, Erk1/2 pERK1/2, STAT1, pSTAT1, Beclin-1 and LC3 were procured from Cell Signaling Technologies, USA. ELISA kits for IFN-ϒ, IL-12p40, IL-6, IL-10 and TGF-β were from e-Biosciences. Recombinant mouse GM-CSF was from R&D Systems (Minneapolis, MN). Dichloro-dihydro-fluorescein diacetate (DCFH-DA was procured from Sigma Chemical Co (St. Louis MO). SUMOylation specific inhibitor 2-D08 was purchased from CalBiochem (St. Louis, MO). GFP-BCG was a kind gift from Prof. Jaya S Tyagi, All India Institute of Medical Sciences, New Delhi, India [13].

### Generation of BMDCs

Bone Marrow derived Dendritic Cells (BMDCs) were differentiated with 15 ng/ml mouse GM-CSF as described before [9, 14–16]. Briefly, the tibia and femur were flushed out from 5–6 BALB/c mice and bone marrow were pooled. Lymphocytes and I-A^+^ cells were depleted following MACS. Cells were cultured in RPMI-1640 medium containing 10% FBS, 0.05M 2-mercaptoethanol, 1mM sodium pyruvate plus 15ng/ml GM-CSF. We have shown that this method gives a homogenous population that is 99% DCs with negligible monocytes or macrophages [6, 9, 15, 17, 18]. The DCs were then stimulated/infected as indicated. Each experiment was done a minimum of three times.

### Experiments with macrophages

Human monocytic cell line (a kind gift from Dr. Dhiraj K Singh from ICGEB) maintained in RPMI-1640 media supplemented with 10% FBS was used to culture cells. THP1 monocytes were differentiated into macrophages via incubation with Phorbol-12-myristate-13-acetate (PMA) with 50ng/ml of PMA for 16 hours [19].

### Recombinant protein expression

Rv3416 was recombinantly expressed as described previously [6, 20]. Briefly, Rv3416 was cloned in pQE31 (Qiagen) vector and expressed as a His-tagged recombinant protein in *E*. *coli* following standard procedures. The expression of Rv3416 was observed in inclusion bodies. Protein expressed as inclusion bodies was purified by batch method with Nickel affinity column under denaturing conditions with buffers containing urea, as per the manufacturer’s instructions (Qiagen). Excess urea was removed by conventional-step dialysis, with reducing concentrations of urea in 10 mM NaH2PO4 buffer (pH 8). Endotoxin levels were estimated and found to be less than 0.05 ng/ml.

### T cell enrichment and processing

Four to six weeks old female BALB/c mice were immunized with 3 million BCG or 15 μg/ml Rv3416 intraperitoneally for one week and thereafter T lymphocytes from spleen were enriched by MACS, as described earlier [21, 22]. Cells were incubated with anti-CD11c, anti-CD11b, anti-I-A^+^, and anti-B220^+^ microbeads to remove DCs, macrophages, MHC-II^+^ cells, and B lymphocytes, respectively. The purity of the enriched T cells was 98%, as ascertained by surface staining for CD90.2. The percentage of I-A^+^ cells was 0.05%. T cells were co-cultured with either Rv3416 or BCG stimulated BMDCs for 48h. For some experiments, DC:T cell co-culture was treated with 3mM EDTA for 10min and T cells were negatively selected by MACS following incubation with CD11c^+^-microbeads. Enriched T cells were incubated with J774 mouse macrophages infected with GFP-expressing BCG.

### Metabolic viability assay

10,000 BMDCs were seeded in 96 well plates and treated with 25μM 2-D08 for 24h. MTT at a concentration of 5mg/ml was added to the culture well for 4h. Formazan crystals formed during the incubation period were then dissolved in DMSO. The absorbed intensity was recorded at 570nm with a reference wavelength of 620nm. All experiments were performed in triplicates.

### Colony forming Unit (CFU) monitoring

BMDCs or THP-1 derived macrophages were incubated with 25μM 2-D08 for 1h followed by infection with 10 MOI BCG for 72h. Cells were lysed with 0.05% SDS and plated in serial dilution on 7H11 agar plates. Two-three weeks later plates were scored for CFU.

### Intracellular reactive oxygen (ROS) species measurement

Intracellular ROS levels were measured using redox sensitive dye DCFH-DA by flow cytometry as described earlier [9, 21, 22]. BMDCs or THP-1 derived macrophages were incubated with 25μM 2-D08 for 1h followed by stimulation with 15μg/ml Rv3416 for 1h. Thirty minutes before the end of incubation cells were incubated with DCFH-DA (10μM) at 37°C in the dark. Cells were washed with 1xPBS with pulse spin and acquired in FACS Calibur (BD Biosciences). The data were plotted and analyzed using CellQuest Pro software.

### Measurement of nitric oxide (NO) levels

BMDCs or THP-1 human macrophages were incubated with 25μM 2-D08 for 1h followed by stimulation with 15μg/ml Rv3416 for 24h. Nitric oxide level was monitored by Griess reagent method using spectrophotometer. This assay determines the nitric oxide based on the enzymatic conversion of nitrate to nitrite by nitrate reductase. The reaction is followed by a colorimetric detection of nitrite as an azo dye product of Griess reagent. Sodium nitrite was used as a standard. Equal amount of NEDA (1 mg/ml) and sulfanilic acid solution (1% in 5% phosphoric acid (10 mg/ml) was added to make Griess reagent. For nitrite measurement, equal amount of sample and Griess reagent was added. The plate was incubated for 30-45min in the dark at room temperature and absorbance was measured at 540 nm.

### Expression of T cell costimulatory molecules

Expression of co-stimulatory molecules were measured by Flow cytometry. BMDCs or THP-1 derived macrophages were incubated with 25μM 2-D08 for 1h followed by stimulations with 15μg/ml Rv3416 for 24h. The cells were then stained with fluorochrome conjugated anti-mouse or anti-human antibodies to CD40 or PDL-1. Cells were washed with 1xPBS followed by pulse spin as described earlier [23]. Surface fluorescence was assayed by FACS Calibur (BD Biosciences). The data were plotted and analyzed using CellQuest Pro software.

### Biotinylation of antibodies

Antibodies to pp38, pERK, pSTAT1, LC3, Beclin-1, Bax and Cytochrome C were biotinylated using N-hydroxysuccinimide ester Biotin (NHS-biotin) as per standard protocols and as described earlier [9, 19]. Briefly, 2μg of each antibody was mixed in 0.1M sodium carbonate buffer (pH 9.5). NHS-Biotin (Sigma) at a concentration of 22mg/ml was then added to the total volume. After, gentle mixing contents were incubated at room temperature for 4h. The reaction mixtures were dialyzed in 1xPBS (pH 7.4) overnight at 4°C to remove excess NHS-Biotin. After dialysis Streptavidin-FITC or Streptavidin-PE was added to the biotinylated antibody and incubated for 20min at room temperature.

### Confocal microscopy

#### For key signaling molecules, autophagy and apoptosis

BMDCs were seeded on UV treated coverslips in 12 well culture dishes. BMDCs were incubated with 25μM 2-D08 for 1h followed by stimulations with 15μg/ml Rv3416 for 1h to monitor the expression levels of pp38, pERK and pSTAT1; and for 24h to monitor the expression levels of Beclin-1, Bax, Cytochrome C. At the end of incubation cells were fixed with 4% paraformaldehyde in 1xPBS for 20min and washed with 1xPBS. Cells were later permeabilized with permeabilization buffer (0.5%BSA+0.2% saponin) for 15min and washed with 1xPBS. Following this, cells were incubated with the desired biotinylated-streptavidin-FITC conjugated antibody for 4h. Cells were washed again with 1xPBS and mounted with DAPI. Confocal imaging was performed using Nikon C2 (Minato, Tokyo, Japan) laser scan confocal microscope with 60x magnification. Images were analyzed using ImageJ Software.

#### For LC3 puncta

BMDCs were incubated with 25μM 2-D08 for 1h followed by stimulations with 2.5MOI of GFP-BCG for 24h. At the end of incubation cells were fixed with 4% paraformaldehyde in 1xPBS for 20min and washed with 1xPBS. Cells were later permeabilized with permeabilization buffer (0.5%BSA+0.2% saponin) for 15min and washed with 1xPBS. Following this, cells were incubated with biotinylated-streptavidin-PE-conjugated LC3 antibody overnight at 4°C. Cells were washed again with 1xPBS and mounted with DAPI. LC3 signals were observed by confocal imaging performed using Nikon C2 (Minato, Tokyo, Japan) laser scan confocal microscope with 60x magnification. Images were analyzed using the NIS Elements AR software.

#### For intracellular survival of mycobacteria in BMDCs

BMDCs were incubated with 25μM 2-D08 for 1h. For some experiments, BMDCs were transfected with either control siRNA or siRNAs to *Sumo1* or *Ubc9* as described below. Cells were then infected with 10 MOI GFP-expressing BCG for 72h. Cells were washed with 1xPBS and mounted on slides with DAPI.

#### For monitoring effector function of T cells

This was carried out essentially as described recently (22). Briefly, 2-D08 treated or *Sumo1* or *Ubc9* knockdown BMDCs were infected with 2.5 MOI BCG for 24. These BMDCs were co-cultured with BCG primed T cells for 48h. In parallel, J774 mouse macrophages were infected with 10 MOI GFP-expressing BCG for 24h. Following this T cells enriched from DC:T cell co-culture were layered onto GFP-BCG infected macrophages and incubated for 72h. Confocal imaging was performed with Nikon C2 laser scan confocal microscope with 60x magnification. Data were analyzed using NIS Elements AR software.

### siRNA mediated knockdown of genes in the SUMOylation pathway

siRNA mediated knockdown of specific genes was carried out as described earlier [6, 9, 21, 23]. Briefly, 3 x10^6^ bone marrow precursor cells from naive mice were transfected with 60 pmoles of siRNAs against Control, *Sumo1* and *Ubc9*. After 4h RPMI-1640 medium containing 20% FBS, 0.05M 2-mercaptoethanol, 1mM sodium pyruvate and 15ng/ml mouse GM-CSF was added and incubated for 72h. Cells were stimulated or infected as indicated above and processed.

### Estimation of cytokines

Cytokines were measured as described previously [22, 24, 26]. Briefly, BMDCs were treated with 25μM 2-D08 for 1h followed by stimulations with 15μg/ml Rv3416 for 24h. Culture supernatants were analyzed for indicated cytokines by sandwich ELISA. For some experiments, BMDCs were treated with 25μM 2-D08 for 1h followed by stimulation with 15μg/ml Rv3416 for 24h. These BMDCs were then co-cultured with Rv3416 primed T cells for 48h. Culture supernatants of BMDCs or DC:T cell co-culture were analyzed for the levels of IFN-γ, IL-6, IL-12p40, IL-10 or TGF-β by sandwich ELISA protocol, as recommended by the manufacturer and as described earlier [22]. Briefly, ELISA plates were coated with 100μl/well of capture antibody overnight at 4°C. Plates were washed with 1xPBST and wells were blocked with ELISPOT diluent for 1h at room temperature. Plates were washed with 1xPBST and wells and coated with samples and standards overnight at 4°C. Plates were washed with 1xPBST and wells were coated with 100μl/well of detection antibody for 1h at room temperature. Plates were washed with 1xPBST and coated with 100μl well of diluted avidin HRP. Plates were sealed and incubated at room temperature for 30min. Wells were washed with PBST and incubated with 100μl/well of 1x TMB (3,3’5,5’-Tetramethylbenzidine) at room temperature for 15min. 50μl /well of stop solution was added to the wells and plates were read at 450nm. The sensitivity ranges of cytokines were 31.2 to 4000pg/ml. Quantification was done against a standard curve obtained for individual cytokine standards provided by the manufacturer.

### Western blotting

BMDCs or THP-1 derived macrophages were treated with 25μM 2-D08 for 1h followed by stimulations with 15μg/ml Rv3416 for indicated time points. For some experiments BMDCs or THP-1 macrophages were infected with the indicated MOI of BCG. Total cell extracts were prepared using 1x RIPA Buffer. 30μg of total cell extracts was resolved on SDS-PAGE and subsequently transferred onto nitrocellulose membrane. Blots were then probed with antibodies to SUMO1, pp38, pERK, pSTAT1, p38, ERK, STAT1, LC3, Beclin-1, Bax or Cytochrome C, followed by HRP labelled secondary antibody. Blots were later developed by chemiluminescence using luminol reagent. A parallel set of samples was run separately and probed either GAPDH or β-actin as loading control as described previously [19].

### Co-immunoprecipitation

Total cell extract was prepared as indicated above. 100μg of extract were incubated with antibodies against the protein of interest (pp38, pSTAT1, LC-3, Beclin-1 and Cytochrome C) at 4°C on a rocker. Followed by addition of protein A/G agarose beads for overnight at 4°C on a rocker. The beads were washed and resolved on SDS-PAGE, followed by transfer to nitrocellulose membrane. Blots were then probed for the SUMO1 [24].

### Statistics

Statistical analysis was performed using multiple measures ANOVA, followed by the Bonferroni’s multiple comparison post hoc test to determine difference among various groups by GraphPad Prism 8 software (San Diego, CA, USA). A two tailed Student’s t-test was also performed for experiments containing two experimental groups by GraphPad Prism 8 software (San Diego, CA, USA). Values of P<0.05 were considered as significant. Mean Fluorescence Intensities of each group were used for comparison and applied to the statistical test performed. (*) denotes statistical significance at *P<0.05, **p<0.01, ***p<0.001, ****p<0.0001 and ns denotes non-significant. For confocal images fluorescence from multiple fields for the mentioned groups was calculated either using ImageJ or by ROI statistics of NIS Elements Analysis Software of 20 fields.

## Results

### Rv3416 and *M*. *bovis* BCG induces SUMOylation of proteins and inhibiting SUMOylation reduces intracellular bacterial burden

**Fig 1:**
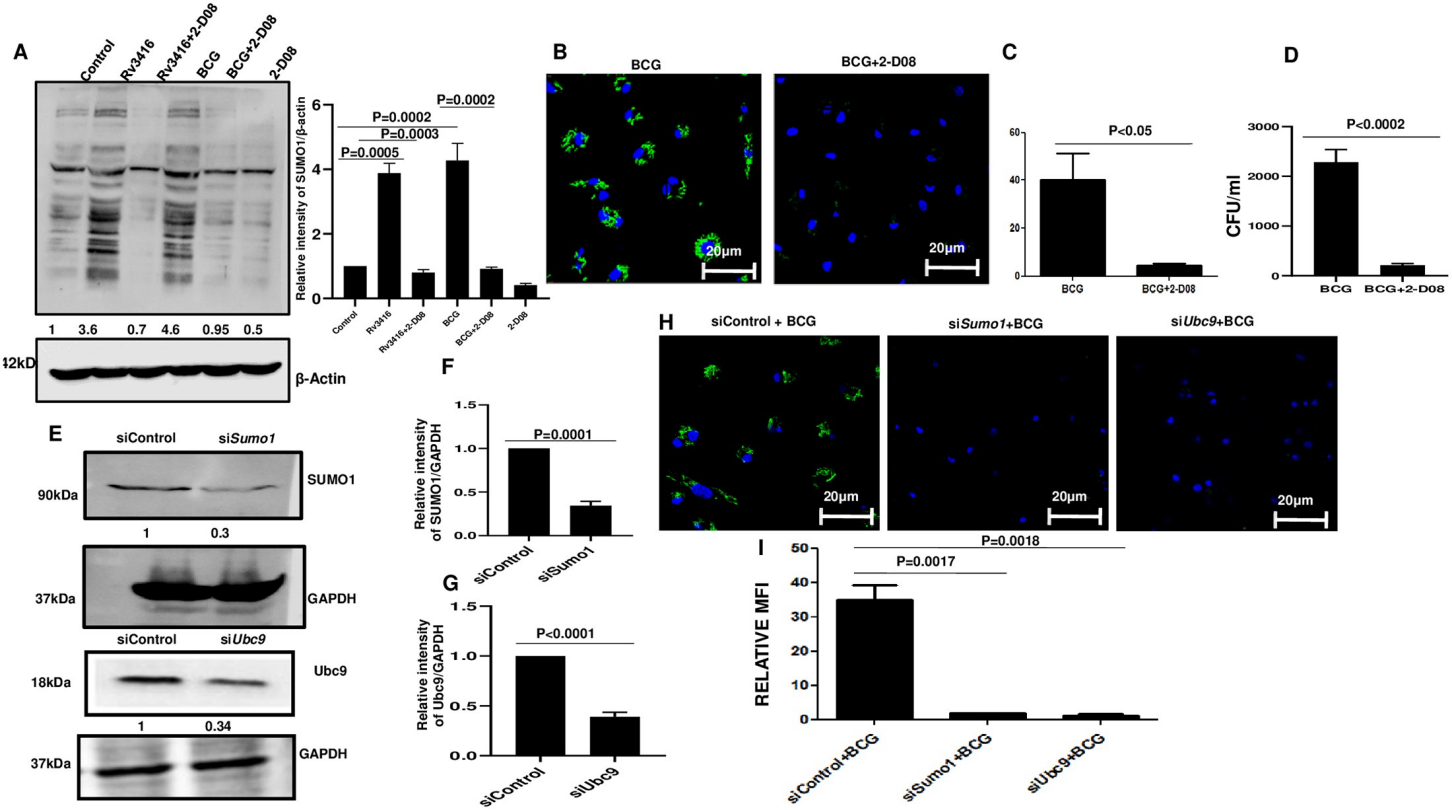
Rv3416 and *M*. *bovis* BCG induces SUMOylation of proteins and inhibiting SUMOylation reduces intracellular bacterial burden. For Panels A BMDCs were incubated with 25μM 2-D08 for 1h followed by stimulations with 15μg/ml Rv3416 or 2.5MOI BCG for 24h. 30μg total cell extract was western blotted for SUMO1. β-actin level was used as a loading control. Bar chart next to Panel A represents the quantification of each lane. For Panel B, BMDCs were incubated with 25μM 2-D08 for 1h followed by infection with 10 MOI GFP-BCG for 72h. Bacterial survival was monitored by confocal microscopy. In Panel B, blue indicates nucleus stained with DAPI and green indicates GFP-BCG. Data from one of three independent experiments are shown (n = 3). The MFI numbers in the bar graphs of Panel C represents the Mean Intensity (of FITC channel) for all the bacteria present in multiple fields for the mentioned groups as calculated by ROI statistics of NIS Elements Analysis Software of 20 fields of Panel B. Paired Student’s t test was performed with 95% confidence interval. For Panel C, P value between groups BCG and BCG+2-D08 is P<0.05. For Panel D, BMDCs were incubated with 25μM 2-D08 for 1h followed by infection with 10MOI BCG for 72h. Serial dilutions of cell lysates were scored for CFU. Data represents mean ± SD of three independent experiments (n = 3). For Panel D, P value between groups BCG and BCG+2-D08 is P<0.0002. Panel E shows knockdown efficiency of siRNAs against either *Sumo1* or *Ubc9*. Total cell extract was western blotted and probed for levels of SUMO1 and Ubc9. GAPDH levels were used as a loading control. Data from one of two independent experiments is shown (n = 2). Panels F& G represent intensities of specific bands plotted as a function of the band intensity of the corresponding loading control. For Panel F, P value between groups siControl and siSUMO1 is P = 0.0001. For Panel G, P value between groups siControl and siUBC9 is P<0.0001. Panel H shows GFP-BCG levels in BMDCs following knockdown of either *Sumo1* or *Ubc9*. Blue indicates nucleus stained with DAPI and green indicates the GFP labelled BCG. The MFI numbers in the bar graphs of Panel I represents the Mean Intensity (of FITC channel) for all the bacteria present in multiple fields for the mentioned groups as calculated by ROI statistics of NIS Elements Analysis Software of 20 fields of Panel H. Data from one of three independent experiments are shown (n = 3). ANOVA with Bonferroni’s post hoc test was performed with 95% confidence interval For Panel I, P value between groups BCG and siSUMO1+BCG is P = 0.0017; between groups BCG and siUBC9+BCG is P = 0.0018.

To begin with we first investigated if Rv3416 stimulation or *M*. *bovis* BCG (hereafter BCG) infection results in changes in SUMOylation levels of cellular proteins in BMDCs. We previously identified several mycobacterial proteins that are expressed inside infected macrophages as a function of time. One such protein was Rv3416, a mycobacterial transcription factor [21]. Functional characterization of this protein showed that despite being a transcription factor, it still mediated many immuno-suppressive responses such as downregulation of ROS, downregulation of pro-inflammatory cytokines, inhibition of autophagy [6, 9]. Rv3416 also promoted the higher expression of L-type Voltage Gated Calcium Channel (VGCC) expression on macrophages [20]. We had previously shown that VGCC played a negative role in mediating protective responses to mycobacteria [25]. Further, Rv3416 also mediated autophagy and apoptosis in DCs in a TLR2 and DC-SIGNR1 specific manner [6]. Additionally, Rv3416 also played a crucial role in modulating increased survival of macrophages during *M*. *tb* HIV co-infection [19]. All these studies point towards Rv3416 as a potent immuno-suppressive protein. In the light of the above we envisaged if modulation of SUMOylation in BMDCs might also be a strategy of immune evasion by Rv3416. As shown in Fig 1A stimulation of BMDCs with 15μg/ml Rv3416 or 2.5MOI BCG increased the SUMOylation of several host proteins. To address if the increased SUMOylation upon Rv3416 and BCG stimulation would modulate intracellular bacterial survival, we inhibited the SUMOylation pathway using a pharmacological inhibitor 2-D08 as well as siRNA mediated knockdown of *Sumo1* and *Ubc9*, key genes in the SUMOylation pathway. In order to investigate whether the increased SUMOylation would affect DC activation and effector functions, including intracellular mycobacterial survival, we carried out experiments wherein we inhibited SUMOylation and monitored various responses. To that end we inhibited SUMOylation either using a bio pharmacological inhibitor or using specific siRNAs against the key genes in the SUMOylation pathway.

To begin with we first ensured that treatment of cells with SUMOylation inhibitor 2-D08 had no significant effect on cell viability (S1 Fig). Additionally, we also ensured that BCG is taken up by BMDCs and treatment with 2-D08 had no significant effect on the uptake of BCG (S2 Fig). We also ensured that treatment with 2-D08 significantly inhibited the expression of SUMOylation pathway proteins. As shown in Fig 1A, treatment with 2-D08 effectively and significantly decreased the SUMOylation of proteins in both Rv3416 stimulated and BCG infected BMDCs. Next, we investigated if SUMOylation would modulate the bacterial loads inside cells as described recently [17]. BMDCs were either treated with 2-D08 or transfected with siRNAs against *Sumo1* or *Ubc9* followed by infection with 10MOI GFP labelled BCG for 72h. As shown in Fig 1B, inhibiting SUMOylation significantly decreased the survival of mycobacteria in BMDCs, indicating a key role of SUMOylation in mycobacterial survival inside BMDCs. Fig 1C represents the mean fluorescence intensity of the FITC channel for all the bacteria present in multiple fields. A reduction in bacterial load upon inhibiting SUMOylation was similarly obtained by the CFU method (Fig 1D). Further, siRNA mediated knockdown of *Sumo1* and *Ubc9*, genes which play a key role in the SUMOylation pathway, also decreased bacterial burden in DCs as shown Fig 1H. Knockdown efficiency of siRNAs against either *Sumo1* or *Ubc9* is shown in Fig 1E–1G). This also showed that increased expression of SUMO1 by Rv3416 or BCG could be an evasion strategy to regulate bacterial survival. Fig 1I represents the mean intensity of the FITC channel for all the bacteria present in multiple fields of Panel H. Next, we sought to investigate if similar to BMDCs, Rv3416 and BCG would increase the expression of SUMOylation proteins in macrophages and also result in a reduction in intracellular bacterial survival. To that end human monocyte derived THP-1 macrophages were treated with 2-D08 followed by stimulation with Rv3416 or infection with BCG and changes in the levels of SUMOylated proteins as well as bacterial survival were monitored. As shown in S3 Fig (Panel A-C), both Rv3416 and BCG stimulation increased the SUMOylation of a number of proteins. Although when compared to BMDCs, fewer proteins appear to be SUMOylated in macrophages upon stimulation with either Rv3416 or BCG. Significantly, however, inhibiting SUMOylation inhibited the survival of mycobacteria inside macrophages (S3 Fig Panel D).

### Inhibiting SUMOylation pathway reverses downmodulation of oxidative burst by Rv3416 in BMDCs

**Fig 2:**
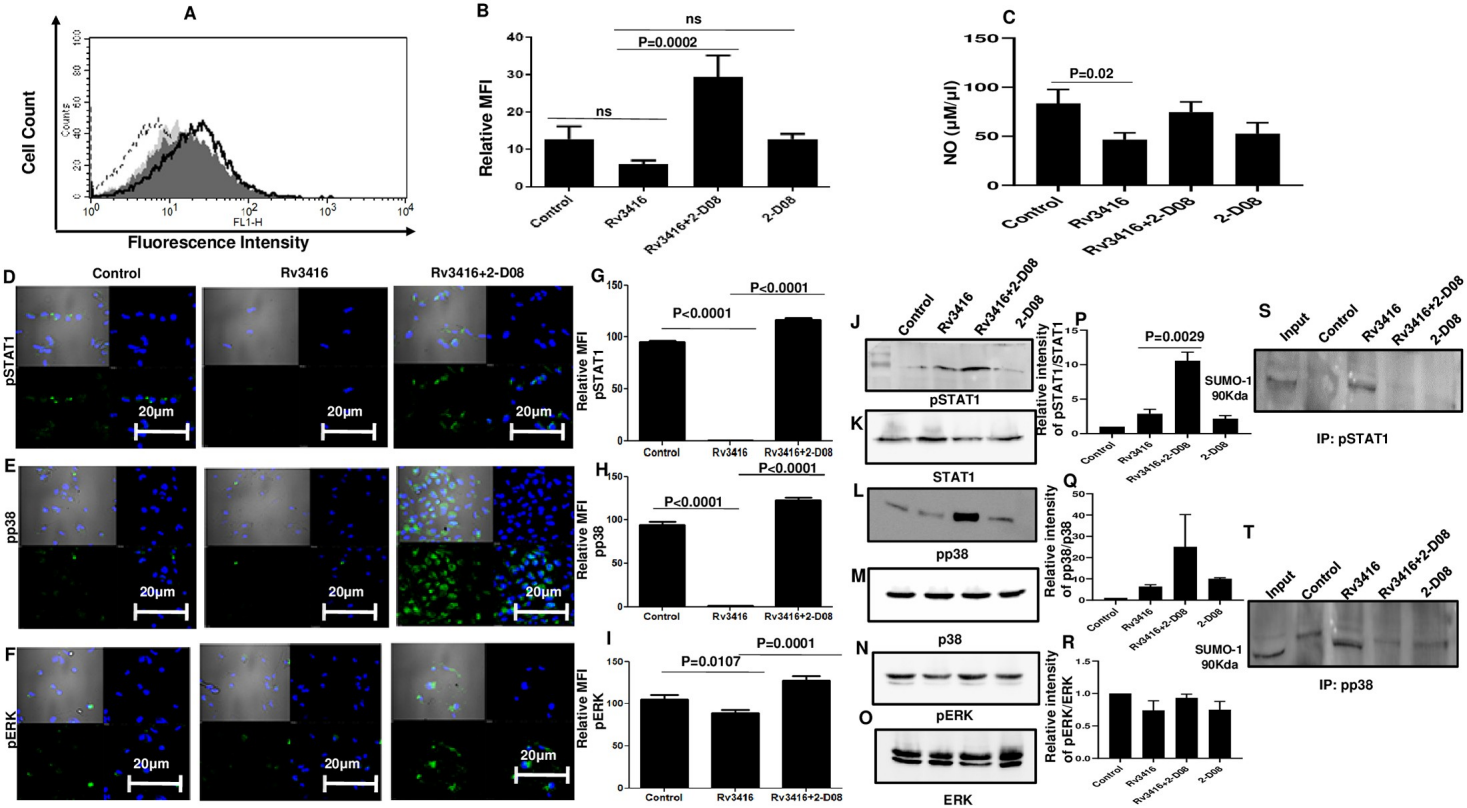
Inhibiting SUMOylation in Rv3416 stimulated BMDCs increases oxidative burst and activation of kinases and transcription factors in BMDCs. For Panel A, BMDCs were incubated with 25μM 2-D08 for 1h followed by stimulation with 15μg/ml Rv3416 for 1h. Oxidative burst was monitored by flow cytometry. Shaded histogram (light grey) represents unstimulated cells, dotted line represents Rv3416 stimulated cells, thick line represent Rv3416 stimulated cells pretreated with 2-D08 and shaded histogram (dark grey) represents cell treated with 2-D08. Multiple measures ANOVA was performed with 95% confidence interval. Bar chart in Panel B represent the Mean Fluorescence Intensities (MFI) of indicated groups as a mean ± SD of three independent experiments (n = 3). ANOVA with Bonferroni’s post hoc test was performed was performed with 95% confidence interval. For Panel B, P value between groups Rv3416 and Rv3416+2-D08 is P = 0.0002. For Panel C BMDCs were incubated with 25μM 2-D08 for 1h followed by stimulation with 15μg/ml Rv3416 for 24h. Nitric oxide level was monitored by Griess reagent method using spectrophotometer. Multiple measures ANOVA was performed with 95% confidence interval Data from mean ± SD of three independent experiments is shown. P value between groups Control and Rv3416 is P = 0.02. In Panels D to F, BMDCs were incubated with 25μM 2-D08 for 1h followed by stimulation with 15μg/ml Rv3416 for 1h. Cells were stained with biotinylated-Streptavidin-FITC conjugated antibodies to the indicated molecules and their expression level was monitored by confocal microscopy. Blue indicates nucleus stained with DAPI and green indicates staining with biotinylated antibodies conjugated to Streptavidin FITC. Top left panel in each image depicts TD, top right panel depicts staining for nucleus, bottom left panel depicts staining for the indicated molecule and bottom right panel depicts the merged image of blue and green stains. Bar charts in Panels G to I represent the mean Fluorescence Intensity of 20 fields of three independent experiments as measured by ImageJ software of the Panels D, E and F, respectively. ANOVA with Bonferroni’s post hoc test was performed with 95% confidence interval. For Panel G, P value between groups Control and Rv3416 is P<0.0001; between groups Rv3416 and Rv3416+2-D08 P<0.0001. For Panel H, P value between groups Control and Rv3416 is P<0.0001; between groups Rv3416 and Rv3416+2-D08 is P<0.0001. For Panel I, P value between groups Control and Rv3416 is P = 0.0107; between groups Rv3416 and Rv3416+2-D08 is P = 0.0001. In Panel J to O, BMDCs were incubated with 25μM 2-D08 for 1h followed by stimulation with 15μg/ml Rv3416 for 1h. Total cell extract was western blotted for pSTAT1, pp38, pERK. Blots for STAT1, p38 or ERK1/2 represent the respective loading controls. Data from one of three independent experiments is shown (n = 3). Panel P to R represents intensities of specific bands plotted as a function of the band intensity of the corresponding loading control. ANOVA with Bonferroni’s hoc post test was performed with 95% confidence interval. For Panel P, P value between groups Rv3416 and Rv3416+2-D08 is P = 0.0029. For Panel S and T total cell extract was co-immunoprecipitated with indicated molecules followed by western blotting with SUMO1 antibody.

We next characterized the critical mechanisms by which SUMOylation would bring about effective reduction in mycobacterial survival in BMDCs. To that end, in the next set of experiments we monitored the role of SUMOylation in the activation of key signaling molecules and key defense responses that play crucial roles during mycobacterial infection. We first investigated the role of SUMOylation in mediating reactive oxygen burst in response to Rv3416 stimulation. We had earlier shown that Rv3416 negatively modulated ROS in BMDCs and macrophages [26, 27]. BMDCs were incubated with SUMOylation inhibitor 2-D08 for 1h followed by Rv3416 stimulation for 1h and levels of ROS were monitored by flow cytometry. As shown in (Fig 2A) and consistent with our earlier report, Rv3416 downmodulated ROS production. However, inhibiting SUMOylation restored ROS production in BMDCs (Fig 2A). In Fig 2B represent the Mean Fluorescence Intensities (MFI) of indicated groups of Fig 2A. Similar results were obtained with macrophages, wherein inhibiting SUMOylation significantly increased ROS levels upon Rv3416 stimulation (S4 Fig Panel A and B). These results show that Rv3416 mediated downmodulation of ROS production involved the SUMOylation pathway.

Several studies demonstrated the role of nitric oxide (NO) in anti-microbial activity via enhanced transcription of NOS gene [28]. In *M*. *tb* infected mice, it has been shown that NO is essential in killing bacteria by phagocytes. Our earlier study showed that Rv3416 downmodulated the NO levels in macrophages [25]. Therefore, next we monitored the NO level in BMDCs as well as macrophages. Consistent with our earlier report, Rv3416 downmodulated NO production. However, inhibiting SUMOylation restored NO production in BMDCs (Fig 2C) as well as macrophages (S4 Fig Panel C).

### Inhibiting SUMOylation pathway in Rv3416 stimulated BMDCs increases the activation of key kinases and transcription factors

It is well documented that Mitogen Activated protein Kinase (MAPK) proteins and transcription factors regulate inflammatory responses to mycobacteria [9]. pERK and pp38 are MAPK pathway proteins that are required for BMDCs maturation and function [29–31], while pSTAT1 plays a critical role in apoptosis and is a key transcription factor that regulates inflammatory responses [31]. We therefore, monitored the role of SUMOylation in regulating the activation status of these molecules. As shown in Fig 2D to 2F and 2J to 2O stimulation of BMDCs with Rv3416 had no appreciable change in the activation of either pSTAT1, pp38 or pERK. However, inhibiting SUMOylation prior to antigenic stimulation significantly enhanced expression levels of pSTAT1, pp38 and pERK. This indicated that Rv3416 enhanced the expression of SUMOylation proteins led to inhibition of MAPK and STAT1 activation. The MFI numbers in the bar graphs of Fig 2 Panel G to I represent the Mean Fluorescence Intensity of the indicated groups of Fig 2D to 2F, respectively. Fig 2P to 2R represent quantitative analysis of indicated group of Fig 2J to 2O.

We next addressed whether, during Rv3416 stimulation pSTAT1, pp38 or pERK are SUMOylated. To that end total cell extract was co-immunoprecipitated using pSTAT1 or pp38 or pERK and western blotted for SUMO1. As shown in S2 Fig and Fig 2T, pSTAT1 and pp38 were found to be associated with SUMO1. S6 Fig shows the reverse immune-precipitation, wherein SUMO1 was immune-precipitated followed by Western blotting for pSTAT1 and pp38. wherein However, no association of pERK was detected. This indicated that pSTAT1 as well as pp38 could be targets of SUMO1.

### Inhibiting SUMOylation prior to Rv3416 stimulation modulates the expression of co-stimulatory molecules on BMDCs

**Fig 3:**
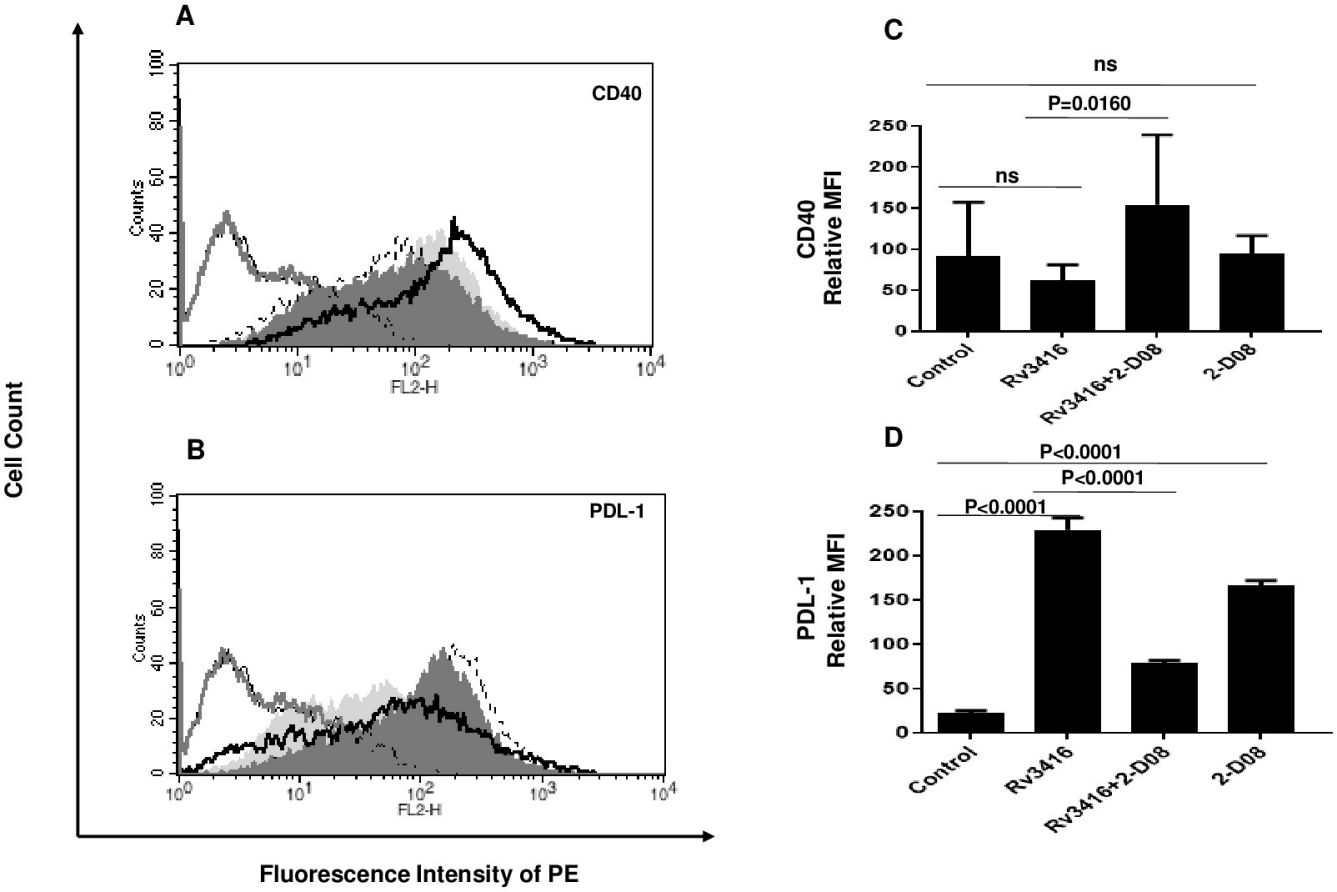
Inhibiting SUMOylation in Rv3416 stimulated BMDCs modulates the surface expression of co-stimulatory molecules. For Panels A and B, BMDCs were incubated with 25μM 2-D08 for 1h followed by stimulation with 15μg/ml Rv3416 for 24h. Surface densities of indicated molecules were monitored by flow cytometry. Thick grey line represents isotype PE, thin black line represents unstained cells, Shaded histogram (light grey) represents unstimulated cells, dotted line represents Rv3416 stimulated cells, thick black line represents Rv3416 stimulated cells pretreated with 2-D08 and shaded histogram (dark grey) represents cells treated with 2-D08. In Panels C and D, bar charts represent Mean Fluorescence Intensities (MFI) of indicated groups as a mean ± SD of three independent experiments (n = 3) for Panels A and B, respectively. ANOVA with Bonferroni’s post hoc test was performed with 95% confidence interval. For Panel C, P value between groups Rv3416 and Rv3416+2-D08 is P = 0.0160. For Panel D, P value between groups Control and Rv3416 is P<0.0001; P value between groups Rv3416 and Rv3416+2-D08 is P<0.0001.

A primary function of DCs is the processing and presentation of antigens to T cells that ensures a productive T cell response. This requires high expression of co-stimulatory molecules [17, 25]. Therefore, we measured the expression levels of co-stimulatory molecules CD40 and PDL-1 following SUMOylation inhibition. While CD40 regulates Th1 responses during cognate DC:T cell interactions, PDL-1 induces T cell exhaustion [6, 23, 32]. Rv3416 stimulation of BMDCs decreased the surface expression of CD40 and significantly increased the expression of PDL-1 (Fig 3A and 3B). However, SUMOylation inhibition increased the expression of CD40 with a concomitant decrease in the expression of PDL-1. Similar results were obtained with macrophages wherein inhibiting SUMOylation significantly increased CD40 levels with a concomitant decrease in PDL-1 levels (S5 Fig). These results indicate that SUMOylation may also hamper the generation of productive T cell responses and favor T cell exhaustion.

### Inhibiting SUMOylation pathway prior to Rv3416 stimulation enhances the production of pro-inflammatory cytokines in BMDCs

**Fig 4:**
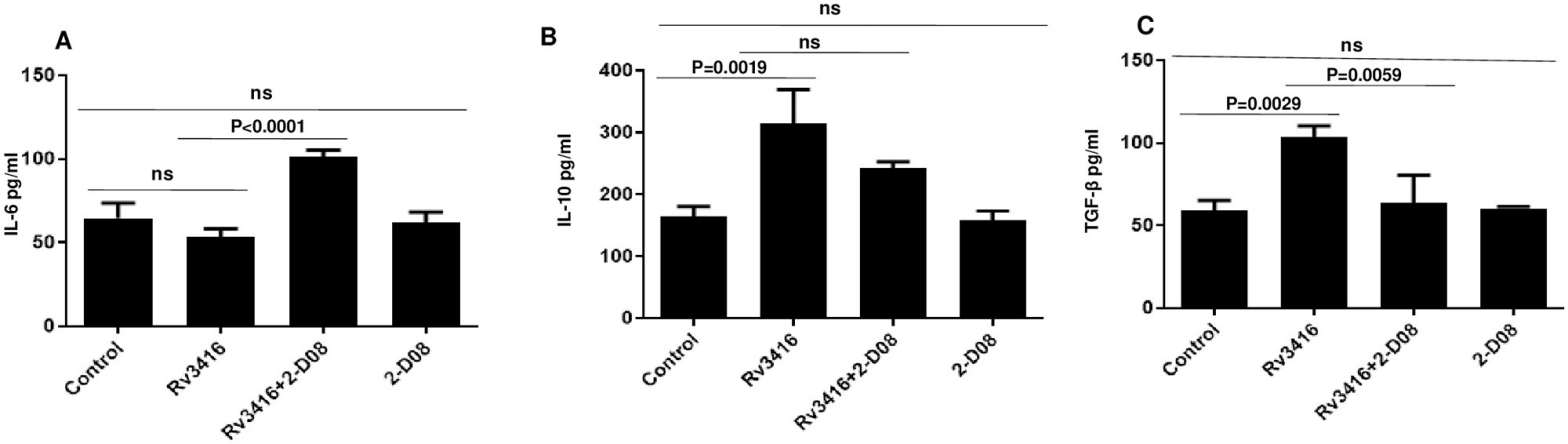
Inhibiting SUMOylation in Rv3416 stimulated BMDCs increases the expression of pro-inflammatory cytokines. For Panel A to C, BMDCs were incubated with 25μM 2-D08 for 1h followed by 15μg/ml Rv3416 for 24h. Levels of indicated cytokines were measured in culture supernatants. Data from mean ± SD of three independent experiments are shown (n = 3). Each group had three replicates. ANOVA with Bonferroni’s post hoc test was performed with 95% confidence interval. For Panel A, P value between groups Rv3416 and Rv3416+2-D08 P<0.0001. For Panel B, P value between groups Control and Rv3416 is P = 0.0019. For Panel C, P value between groups Control and Rv3416 is P = 0.0029; P value between groups Rv3416 and Rv3416+2-D08 is P = 0.0059.

DCs maturation and activation is regulated by pro-inflammatory cytokines that are critical in host defense against mycobacterial infection. BMDCs release these cytokines upon antigenic stimulation which polarizes T cells towards a Th1 phenotype. Therefore, we investigated the role of SUMOylation on modulation of cytokine profiles from DCs upon Rv3416 stimulation. As shown in Fig 4A to 4C, stimulation of BMDCs with Rv3416 decreased IL-6 expression. It however, significantly increased the levels of anti-inflammatory cytokines IL-10 and TGF-β. Inhibition of SUMOylation increased IL-6 levels. More significantly, inhibiting SUMOylation reduced the levels of IL-10 and TGF-β. These results indicate that inhibiting SUMOylation would result in a pro-inflammatory cytokine secretion from BMDCs that would modulate the ensuing T cell responses.

### Inhibiting SUMOylation pathway in Rv3416 stimulated BMDCs increases the expression of autophagy and apoptotic markers

**Fig 5:**
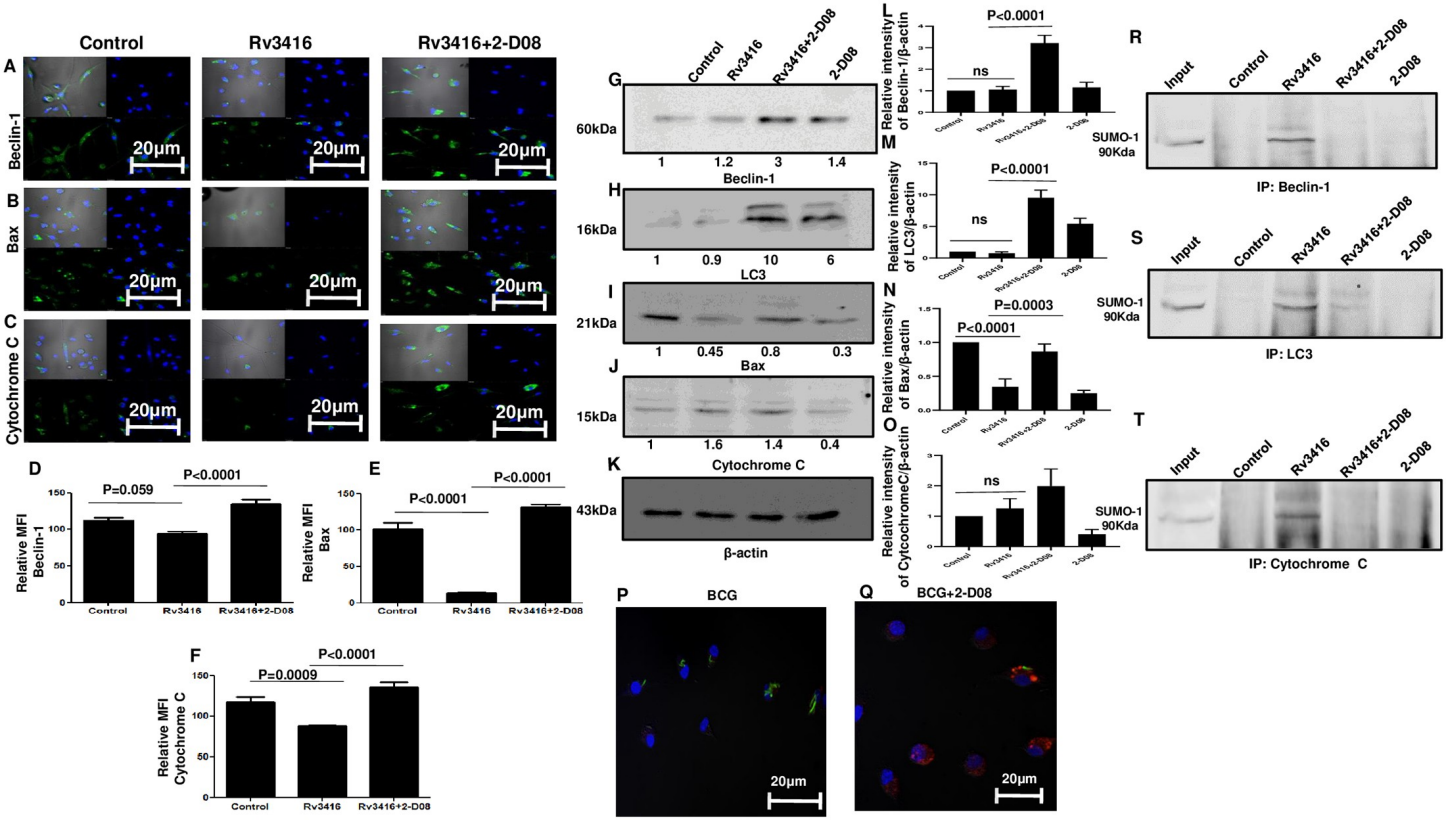
Inhibiting SUMOylation pathway in Rv3416 stimulated BMDCs increases the expression of proteins involved in autophagy and apoptosis. For Panel A to C, BMDCs were incubated with 25μM 2-D08 for 1h followed by 15μg/ml Rv3416 for 24h. Cells were stained with biotinylated-Streptavidin-FITC labelled antibodies to indicated molecules. Expression levels of indicated markers were monitored by confocal microscopy. Blue indicates nucleus stained with DAPI and green indicates staining with antibodies conjugated to Streptavidin FITC. Top left panel in each image depicts TD, top right panel depicts staining for nucleus, bottom left panel depicts staining for the indicated molecule and bottom right panel depicts the merged image of blue and green stains. Mean Fluorescence Intensities (MFI) of 20 fields of three independent experiments as measured by ImageJ software (n = 3). ANOVA with Bonferroni’s post hoc test was performed with 95% confidence interval. In Panels D to F, bar charts represent Mean Fluorescence Intensities (MFI) of indicated groups as a mean ± SD of three independent experiments for Panels A to C, respectively. For Panel D, P values between groups Control and Rv3416 is P = 0.059; P value between groups Rv3416 and Rv3416+2-D08 P<0.0001. For Panel E, P value between groups Control and Rv3416 is P<0.0001; P value between groups Rv3416 and Rv3416+2-D08 is P<0.0001. For Panel F, P value between groups Control and Rv3416 is P = 0.0009; P value between groups Rv3416 and Rv3416+2-D08 is P<0.0001. For Panel G to K, BMDCs were incubated with 25μM 2-D08 for 1h followed by 15μg/ml Rv3416 for 24h. Total cell extract was western blotted for Beclin-1, LC3, Bax, Cytochrome C, or β-actin. Data from one of three independent experiments is shown (n = 3). Panel L to O shows the intensities of specific bands plotted as a function of the band intensity of the corresponding loading control. ANOVA with Bonferroni’s post hoc test was performed with 95% confidence interval. For Panel L, P value between groups Rv3416 and Rv3416+2-D08 is P<0.0001. For Panel M, P value between groups Rv3416 and Rv3416+2-D08 is P<0.0001. For Panel N, P value between groups Control and Rv3416 is P<0.0001; between groups Rv3416 and Rv3416+2-D08 is P = 0.0003. For Panel P and Q, BMDCs were incubated with 25μM 2-D08 for 1h followed by stimulations with 2.5MOI of GFP-BCG for 24h. At the end of incubation cells were fixed with 4% paraformaldehyde in 1xPBS for 20min and washed with the 1xPBS. Cells were later permeabilized with permeabilization buffer (0.5%BSA+0.2% saponin) for 15min and washed with 1xPBS. Following this, cells were incubated with PE conjugated LC3 antibody for overnight at 4°C. Cells were washed again with 1xPBS and mounted with DAPI. LC3 signals were observed by confocal imaging performed using Nikon C2 (Minato, Tokyo, Japan) laser scan confocal microscope with 60x magnification. Images were analyzed using the NIS Elements AR software. For Panel R to T total cell extract was co-immunoprecipitated with indicated molecules followed by western blotting with SUMO1 antibody.

In the next set of experiments, we explored the effects of SUMOylation on apoptosis and autophagy. Mycobacteria modulate autophagy and apoptosis to survive in DCs and macrophages [9, 25]. We reported earlier that Rv3416 inhibits autophagy and apoptosis in DCs [6] and macrophages [19] in order to establish long-term infection. As shown in Fig 5 (Panel A and G) and consistent with our earlier data, stimulation of BMDCs with Rv3416 showed no significant expression of autophagy marker Beclin-1. However, inhibiting SUMOylation significantly increased the levels of Beclin-1. During autophagy LC3 is processed and recruited to the autophagosomal membrane. Similar to Beclin-1, Rv3416 stimulations decreased LC3 levels (Fig 5H). However, inhibiting SUMOylation significantly increased LC3 levels. We also monitored autophagic cells by visualizing LC3 puncta. As shown in (Fig 5P) no significant LC3 puncta were observed in cells infected with GFP expressing BCG. However, inhibiting SUMOylation significantly increased LC3 puncta (Fig 5Q).

Similarly, as shown in Fig 5B, 5C, 5I and 5J) Rv3416 stimulation decreased the levels of pro-apoptotic markers Bax and Cytochrome C. However, inhibiting SUMOylation significantly increased the levels of Bax and Cytochrome C. The data in Fig 5 clearly indicates that SUMOylation inhibits apoptosis and autophagy in Rv3416 stimulated BMDCs and increases the survival of cells, possibly to promote the survival of intracellular mycobacteria.

Similar to signaling molecules, we performed co-immunoprecipitation to investigate whether during Rv3416 stimulation SUMO1 associated with Beclin-1, LC3, Bax and Cytochrome C. As shown in Fig 5R to 5T, Rv3416 increased the association of SUMO1 with Beclin-1, LC3 and Cytochrome but not Bax. S7 Fig shows the reverse immune-precipitation, wherein SUMO1 was immune-precipitated followed by Western blotting for Beclin-1, LC-3 and cytochrome C. These results suggest that Rv3416 not only inhibits the expression autophagy and pro-apoptotic molecules, but also may promote their SUMOylation to possibly prevent their action.

### SUMOylation inhibited BMDCs prime T cell effector function

**Fig 6:**
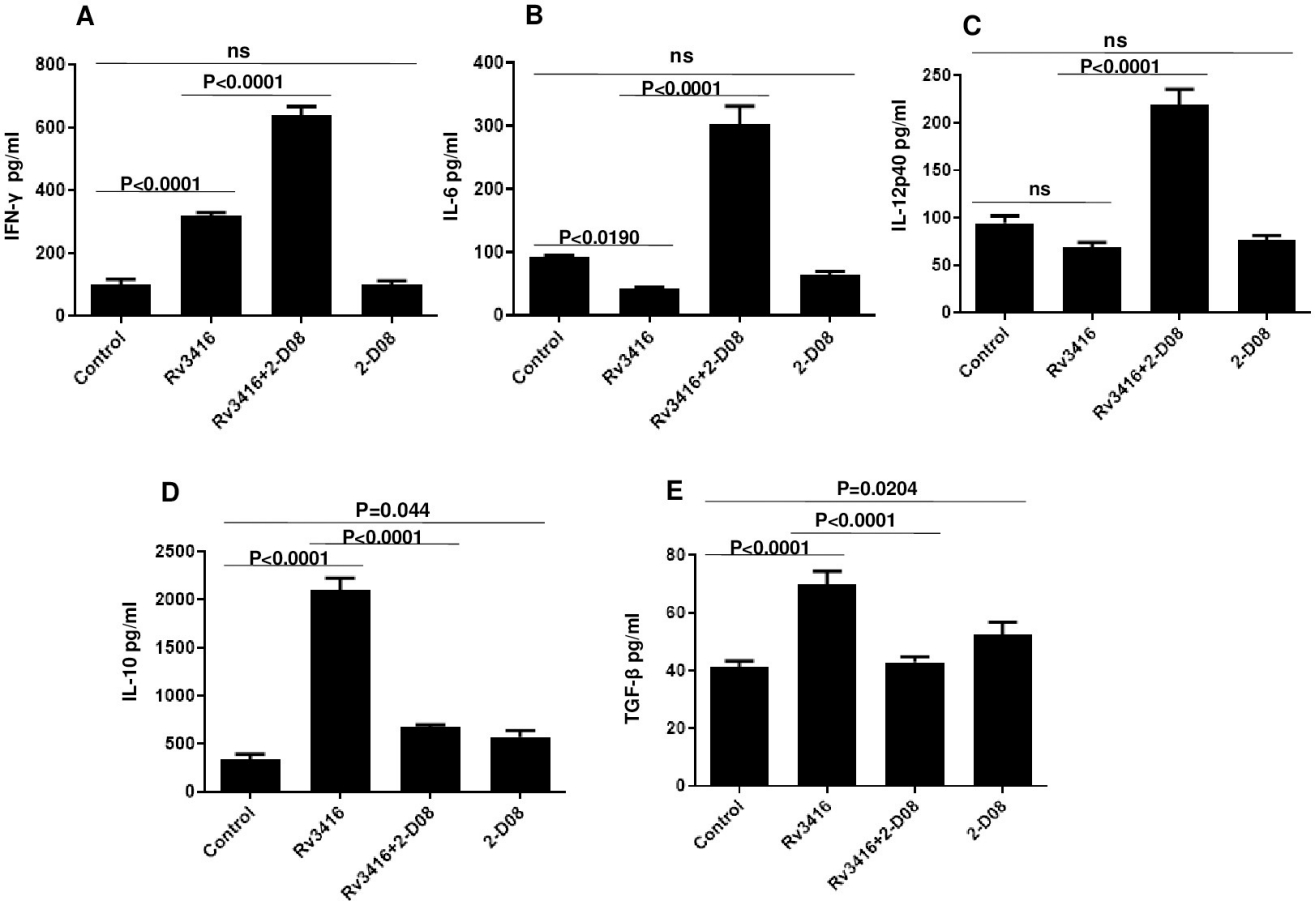
SUMOylation inhibition induces pro-inflammatory responses during DC:T cell cognate interactions. For Panels A-E, BMDCs were incubated with 25μM 2-D08 for 1h followed by stimulation with 15μg/ml Rv3416 for 24h. BMDCs were then co-cultured for 48h with T cells enriched from spleens of Rv3416 primed BALB/c mice. Controls represents naïve T cells primed with 2-D08 treated BMDCs stimulated with 15μg/ml Rv3416 for 24h. Culture supernatants were analyzed for the levels of indicated cytokines by ELISA. Data represents Mean ± SD of three independent experiments (n = 3). ANOVA with Bonferroni’s post hoc test was performed with 95% confidence interval. For Panel A, P value between groups Control and Rv3416 is P<0.0001; P value between groups Rv3416 and Rv3416+2-D08 P<0.0001. For Panel B, P value between Control and Rv3416 is P<0.0190; P value between groups Rv3416 and Rv3416+2-D08 P<0.0001. For Panel C, P value between groups Rv3416 and Rv3416+2-D08 is P<0.0001. For Panel D, P value between groups Control and Rv3416 is P<0.0001; P value between groups Rv3416 and Rv3416+2-D08 P<0.0001. For Panel E, P value between groups Control and Rv3416 is P<0.0001; P value between groups Rv3416 and Rv3416+2-D08 P<0.0001.

Since a major function of DCs is to stimulate productive T cell responses, in the next set of experiments, we investigated the role of SUMOylation in modulating DC induced T cell effector function. We first investigated the cytokine profiles during DC:T cell cognate interaction. To that end, SUMOylation inhibited and Rv3416 stimulated BMDCs were co-cultured with Rv3416 primed T cells for 48h and cytokines were measured in the culture supernatants. As shown in Fig 6, T cells from Rv3416 stimulated BMDCs produced low levels of IL-6 and IL-12p40, although they did produce IFN-γ when compared to unstimulated cells. They, however, produced higher levels of IL-10 and TGF-β. However, inhibition of SUMOylation significantly enhanced the expression levels of IFN-γ, IL-6, and IL-12p40, while effectively decreasing the levels of IL-10 and TGF-β. These results indicated that inhibiting SUMOylation induced a pro-inflammatory cytokine milieu during DC:T cell interactions.

**Fig 7:**
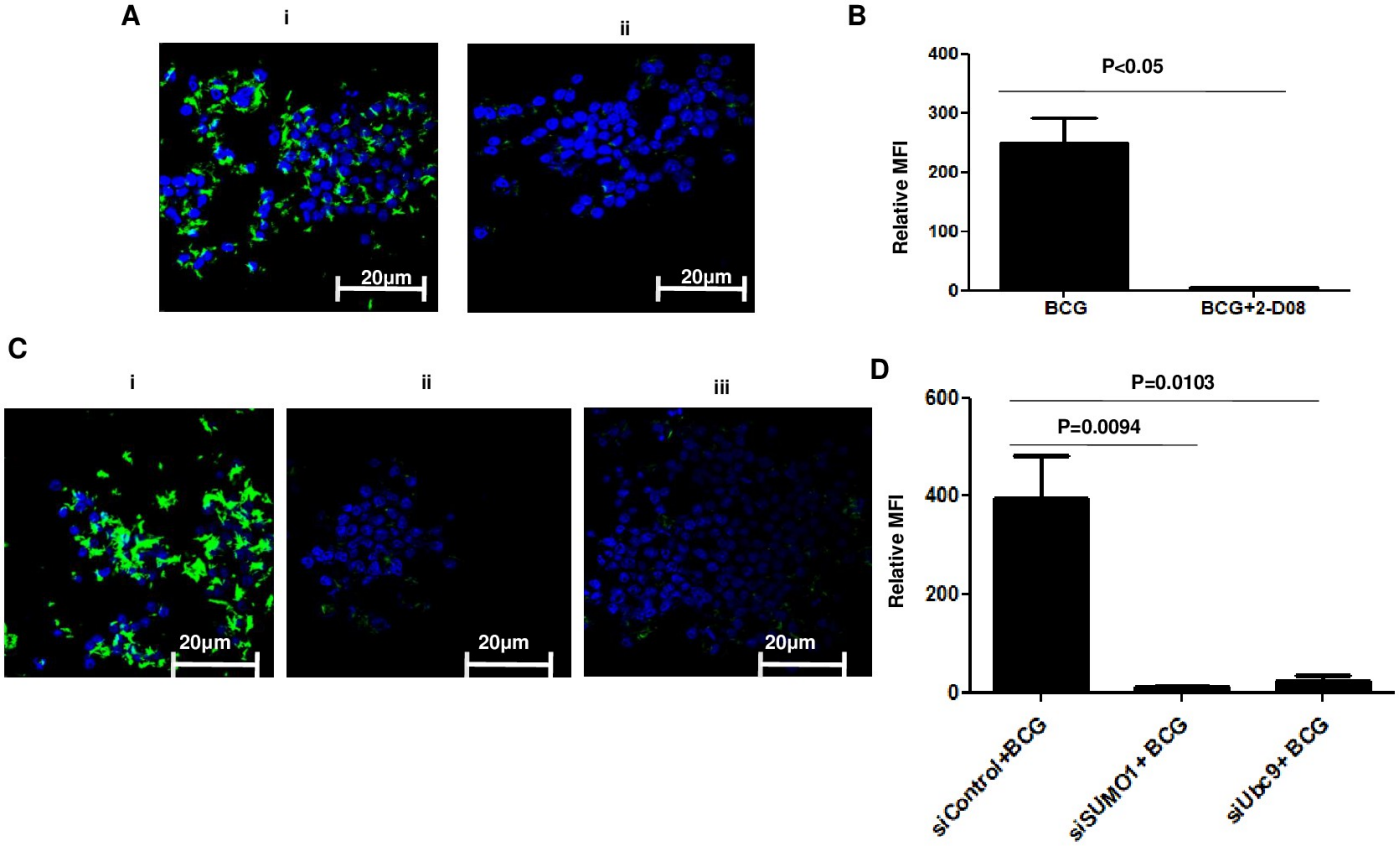
T cells activated by SUMOylation inhibited BMDCs reduce bacterial burden in BCG infected macrophages. BMDCs treated with either 25μM 2-D08 or siRNAs against *siSumo1* or *siUbc9* followed by infection with 10MOI BCG for 24h. BMDCs were then co-cultured with T cells from BCG primed mice for 48h. T cells were then enriched from DC:T cell co-culture and incubated for 72h with J774 murine macrophages (that were earlier infected with 10 MOI GFP-BCG for 24h). Bacterial load was monitored by confocal microscopy. Panel A(i) shows bacterial loads of GFP-BCG in J774 macrophages following addition of T cells that were co-cultured with BCG infected BMDCs (without 2-D08 treatment) and Panel A(ii) shows bacterial loads of GFP-BCG in J774 macrophages following addition of T cells that were co-cultured with BCG infected BMDCs with 2-D08. The MFI numbers in the bar graphs of Panel B represents the Mean Intensity (of FITC channel) for all the bacteria present in multiple fields for the mentioned groups as calculated by ROI statistics of NIS Elements Analysis Software of 20 fields of Panel A. Data from one of three independent experiments is shown (n = 3). Paired Student’s t test was performed with 95% confidence interval. For Panel B, P value between groups BCG and BCG+2-D08 is P<0.05. Panel C shows bacterial loads of GFP-BCG in J774 macrophages following addition of T cells that were co-cultured with BCG infected DCs treated control siRNAs (i), *siSumo1* specific siRNAs (ii) and *siUbc9* specific siRNAs (iii). Data from one of three independent experiments is shown. The MFI numbers in the bar graphs of Panel D represents the Mean Intensity (of FITC channel) for all the bacteria present in multiple fields for the mentioned groups as calculated by ROI statistics of NIS Elements Analysis Software of 20 fields of Panel C. Data from one of three independent experiments is shown (n = 3). ANOVA with Bonferroni’s post hoc test was performed with 95% confidence interval. For Panel D, P value between groups siControl+ BCG and BCG+siSUMO1 is P = 0.0094; P value between groups siControl+ BCG and BCG+siUbc9 is P = 0.0103.

We further extended these observations to investigate the effector functions of SUMOylation inhibited DC-primed T cells as described by us recently [22]. To that end SUMOylation inhibited (with 2-D08) or siRNA knockdown (of *siSumo1* or *siUbc9*) BMDCs were infected with BCG for 24h and later co-cultured with BCG primed T cells for 48h. T cells from the co-culture were enriched and incubated with GFP-BCG infected J774 murine macrophages for 72h. Bacterial load was monitored by confocal microscopy. As shown in Fig 7, T cells primed with SUMOylation inhibited or knockdown BMDCs, effectively helped infected macrophages to significantly reduce the load of BCG burden. These results clearly demonstrated the ability of SUMOylation to mediate T cell effector functions.

## Discussion

For the establishment of successful infection pathogens interfere with host machinery that include many post-translational modifications [7]. Ubiquitination, neddylation and SUMOylation are related pathways that are critical for the host in dealing with various stress responses. These include invasion by viruses and bacteria, dysregulated processes that lead to tumors and cancers [33]. Using an siRNA-based screen we previously identified various genes involved in the cysteine protease and calcium calmodulin pathways that played negative roles in the survival of *M*. *tb* inside BMDCs [6]. One of the genes that played a negative role in mediating protective responses was *Senp8*. SENP family proteins primarily regulate key post-translational processes like neddylation. We earlier reported that neddylation played a suppressive role in regulating various defense responses in BMDCs during mycobacterial infection [9]. Inhibiting neddylation promoted ROS generation, induced pro-inflammatory cytokine responses and regulated phagosome-lysosome fusion. In this study, we explored the roles of SUMOylation, a pathway related to neddylation, during mycobacterial infection in BMDCs. To that end, we first showed that stimulation with Rv3416 or infection with BCG increased the SUMOylation of proteins. Our previous work with Rv3416 had indicated that it is a highly immuno-suppressive protein/antigen of mycobacteria [6, 20, 25, 33]. Therefore, in order to investigate, if increased expression of SUMOylation pathway proteins by Rv3416 was suppressive (or protective) in nature, we inhibited SUMOylation in BMDCs and monitored its effect on key protective responses. We began by showing that stimulation with either a mycobacterial antigen or infection with mycobacteria induced SUMOylation of various proteins. Increased SUMOylation promoted the survival of mycobacteria inside BMDCs, since bio-pharmacological inhibition or knockdown of SUMOylation pathway genes by siRNA effectively attenuated mycobacterial survival in BMDCs. In the next set of experiments, we deciphered the possible and related mechanisms that are modulated by SUMOylation that may help mycobacteria to thwart protective responses mediated by the host. To that end we showed that inhibiting SUMOylation increased ROS and NO responses that were accompanied by increased activation of key signaling molecules pERK and pp38 of the MAPK pathway and pSTAT1 of the JAK/STAT pathway. Surprisingly, Rv3416 also increased the SUMOylation levels of these molecules possibly to prevent their downstream function. pSTAT1 is critical for the activation of IFN-γ responses in DCs and macrophages [34]. Many *M*. *tb* proteins like PPE32, PPE65, PE-PGRS have been shown to inhibit protective responses [35–39]. It has been shown that the 19 kDa lipoprotein of *M*. *tb* inhibits macrophage responses to IFN-γ during *M*. *tb* infection via inhibition of pSTAT1 [39]. Therefore, our results on the role of Rv3416 mediated increased SUMOylation in regulating pSTAT1 activation provides an additional mechanism that may be operative in immune evasion during mycobacterial infection.

Apoptosis and autophagy are known defense mechanisms employed by cells to clear intracellular pathogens [9]. *M*. *tb* is known to inhibit both these processes to establish long term persistent infections. We earlier showed that Rv3416 inhibits both apoptosis and autophagy in DCs and macrophages [6, 19]. We therefore, deciphered the role of SUMOylation in regulating these two processes. Our results show that SUMOylation inhibits apoptosis and autophagy as inhibiting SUMOylation increased the expression levels of pro-apoptotic markers Bax and Cytochrome C as well as autophagy markers LC3 and Beclin-1, thereby decreasing the bacterial load in BMDCs. Recent studies indeed show a cross-regulation between Beclin-1, LC3, Bax, Cytochrome C [40–43].

The data also demonstrate that SUMOylation negatively modulates the expression of T cell co-stimulatory molecules on the surface of BMDCs. This was evident from the increased levels of T cell response promoting molecule CD40 and decreased levels of T cell response inhibitory molecule PDL-1 [23]. The quality of ensuing T cell responses is mediated by the cytokine profiles secreted by DCs (and macrophages) during infection/antigenic stimulation. Our results show that inhibiting SUMOylation promoted a pro-inflammatory cytokine environment during DC:T cell cognate interactions, so much so that T cells activated by SUMOylation inhibited BMDCs, helped mycobacteria infected macrophages to effectively and significantly reduce their bacterial burden. These data showed that SUMOylation in antigen presenting cells had a long-lasting distal effect in clearing intracellular infection. Collectively, the data presented in this study point towards yet another immune evasive mechanism employed by mycobacteria to inhibit immune and defense responses by the host.

## Supporting information

S1 Fig SUMOylation inhibitor 2-D08 does not cause any significant cell death. BMDCs were incubated with 25μM 2-D08 or Rv3416 for 24h. Cell viability was monitored by MTT assay as described in Materials and Methods.
(TIF)

S2 Fig SUMOylation inhibitor 2-D08 does not inhibit internalization of BCG by BMDCs. For Panel A and B BMDCs were seeded on UV treated coverslips in 12 well culture dishes and infected with 10MOI GFP-BCG for 0h (Panel A) and 4h (Panel B). For Panels C and D, BMDCs were seeded on UV treated coverslips in 12 well culture dishes and incubated with 25μM 2-D08 for 1h followed by infection with 10MOI GFP-BCG for 0h (Panel C) and 4h (Panel D). Internalization of GFP-BCG was monitored using Confocal imaging. Data were analysed using NIS Elements Advanced Research Software. Images show Z-stacks of 1.25μm optical sections. Blue indicates staining of nucleus with DAPI.
(TIF)

S3 Fig Rv3416 and BCG induces SUMOylation of proteins pathway proteins in THP-1 macrophages and reduces intracellular bacterial survival. For Panels A THP-1 macrophages were incubated with 25μM 2-D08 for 1h followed by stimulations with 15μg/ml Rv3416 or 2.5MOI BCG for 24h. 30μg total cell extract were western blotted for SUMO1. Data from one of three independent experiments is shown (n = 3). Panel B represents β-actin as loading control. Panels C represent intensities of specific bands plotted as a function of the band intensity of the corresponding loading control. ANOVA with Bonferroni’s post hoc test was performed with 95% confidence interval. For Panel C, P value between groups Rv3416 and Rv3416+2-D08 is P = 0.0040; between groups BCG and BCG+2-D08 is P = 0.001. For Panel D, THP-1 human macrophages were incubated with 25μM 2-D08 for 1h followed by infection with 10MOI BCG for 72h. Serial dilutions of cell lysates were scored for CFU. Data represents mean ± SD of three independent experiments (n = 3). Student’s t test was performed for statistical significance for Panel D. P value between groups BCG and BCG+2-D08 is P = 0.04.
(TIF)

S4 Fig Inhibiting SUMOylation in Rv3416 stimulated THP-1 human macrophages increases oxidative burst and nitric oxide levels. For Panel A, THP-1 human macrophages were incubated with 25μM 2-D08 for 1h followed by stimulation with 15μg/ml Rv3416 for 1h. Oxidative burst was monitored by flow cytometry. In Panel A(a) shaded histogram (light grey) represents unstimulated cells, dotted line represents Rv3416 stimulated cells, while the thin black line depicts unstained cells. In Panel A(b) dark shaded histogram depicts cells treated with 2-D08 only, dotted line depicts Rv3416 treated cells and the thick black line represents Rv3416 stimulated cells pretreated with 2-D08. Multiple measures ANOVA was performed with 95% confidence interval. Bar chart in Panel B represent the Mean Fluorescence Intensities (MFI) of indicated groups as a mean ± SD of three independent experiments (n = 3). For Panel C THP-1 human macrophages were incubated with 25μM 2-D08 for 1h followed by stimulation with 15μg/ml Rv3416 for 24h. Nitric oxide level was monitored by Griess reagent method using spectrophotometer. ANOVA with Bonferroni’s post hoc test was performed was performed with 95% confidence interval. For Panel B, P value between groups Control and Rv3416 is P = 0.002; between group Rv3416 and Rv3416+2-D08 is P<0.0001.
(TIF)

S5 Fig Inhibiting SUMOylation in Rv3416 stimulated THP-1 macrophages modulates the surface expression of co-stimulatory molecules. For Panels A and B, THP-1 human macrophages were incubated with 25μM 2-D08 for 1h followed by stimulation with 15μg/ml Rv3416 for 24h. Surface densities of indicated molecules were monitored by flow cytometry. In Panel A(a) light grey shaded histogram depicts unstimulated cells, thick grey line represents Isotype control and thin black line depicts unstained cells. In Panel A(b) light grey shaded histogram depicts unstimulated cells, dotted line represents Rv3416 stimulated cells, thick black line depicts Rv3416 stimulated cells pre-treated with 2-D08 and dark shaded histogram represents cells treated with 2-D08 only. In Panel B(a) shaded histogram depicts unstimulated cells, thick grey line represents Isotype control and thin black line depicts unstained cells. In Panel B(b) light grey shaded histogram depicts unstimulated cells, dotted line represents Rv3416 stimulated cells, thick black line depicts Rv3416 stimulated cells pre-treated with 2-D08 and dark shaded histogram represents cells treated with 2-D08 only. In Panels C and D, bar charts represent Mean Fluorescence Intensities (MFI) of indicated groups as a mean ± SD of three independent experiments (n = 3) for Panels A and B, respectively. ANOVA with Bonferroni’s post hoc test was performed with 95% confidence interval. For Panel C, P value between groups Rv3416 and Rv3416+2-D08 is P = 0.0056. For Panel D, P value between groups Control and Rv3416 is P = 0.0067; P value between groups Rv3416 and Rv3416+2-D08 is P = 0.0013.
(TIF)

S6 Fig BMDCs were incubated with 25μM 2-D08 for 1h followed by stimulation with 15μg/ml Rv3416 for 1h. Total cell extract was co-immunoprecipitated with SUMO1 followed by western blotting with pSTAT1 or pp38 antibody.
(TIF)

S7 Fig BMDCs were incubated with 25μM 2-D08 for 1h followed by stimulation with 15μg/ml Rv3416 for 1h. Total cell extract was co-immunoprecipitated with SUMO1 followed by western blotting with Beclin-1, LC3 or Cytochrome C antibody.
(TIF)

S8 Fig Represents full size blots for Fig 1 Panels A and E. (TIF)

S9 Fig Represents full size blots for Fig 2 Panel J, Panel K, Panel L, Panel M, Panel N and Panel O, respectively. (TIF)

S10 Fig Represents full size blots for Fig 5 Panel G, Panel H, Panel I, Panel J and Panel K, respectively. (TIF)

S11 Fig Represents full size blots for S3 Fig Panel A and B. (TIF)

S1 File (PDF)
