# Supplementary figures and images for: Mycobacteria modulate SUMOylation to suppresses protective responses in dendritic cells
Source: PLoS One. 2023 Sep 29;18(9):e0283448. doi: 10.1371/journal.pone.0283448 (PMC10540951; doi:10.1371/journal.pone.0283448)

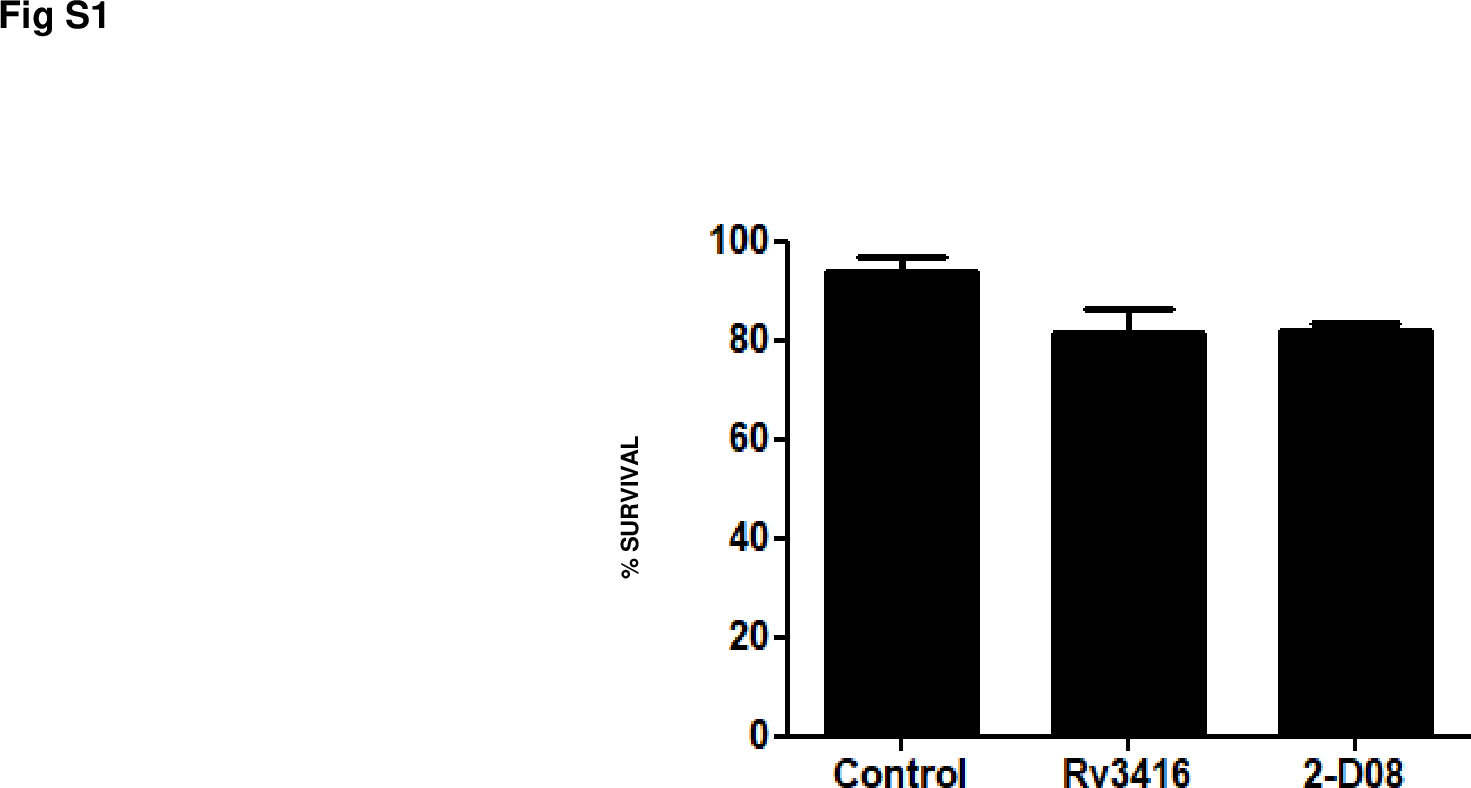

Supplement: S1 Fig — BMDCs were incubated with 25μM 2-D08 or Rv3416 for 24h. Cell viability was monitored by MTT assay as described in Materials and Methods. (TIF) [file pone.0283448.s001.tif]

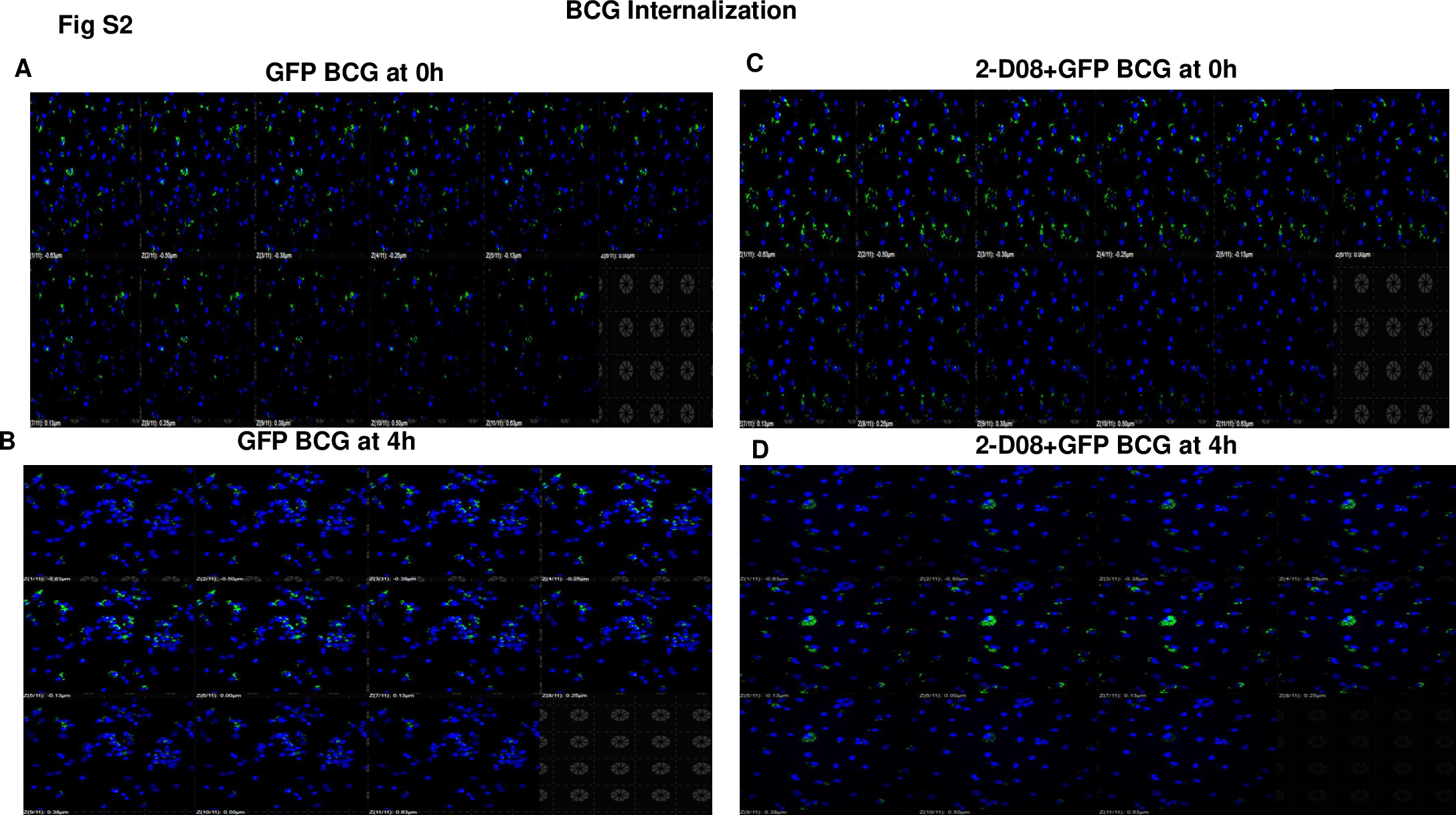

Supplement: S2 Fig — For Panel A and B BMDCs were seeded on UV treated coverslips in 12 well culture dishes and infected with 10MOI GFP-BCG for 0h (Panel A) and 4h (Panel B). For Panels C and D, BMDCs were seeded on UV treated coverslips in 12 well culture dishes and incubated with 25μM 2-D08 for 1h followed by infection with 10MOI GFP-BCG for 0h (Panel C) and 4h (Panel D). Internalization of GFP-BCG was monitored using Confocal imaging. Data were analysed using NIS Elements Advanced Research Software. Images show Z-stacks of 1.25μm optical sections. Blue indicates staining of nucleus with DAPI. (TIF) [file pone.0283448.s002.tif]

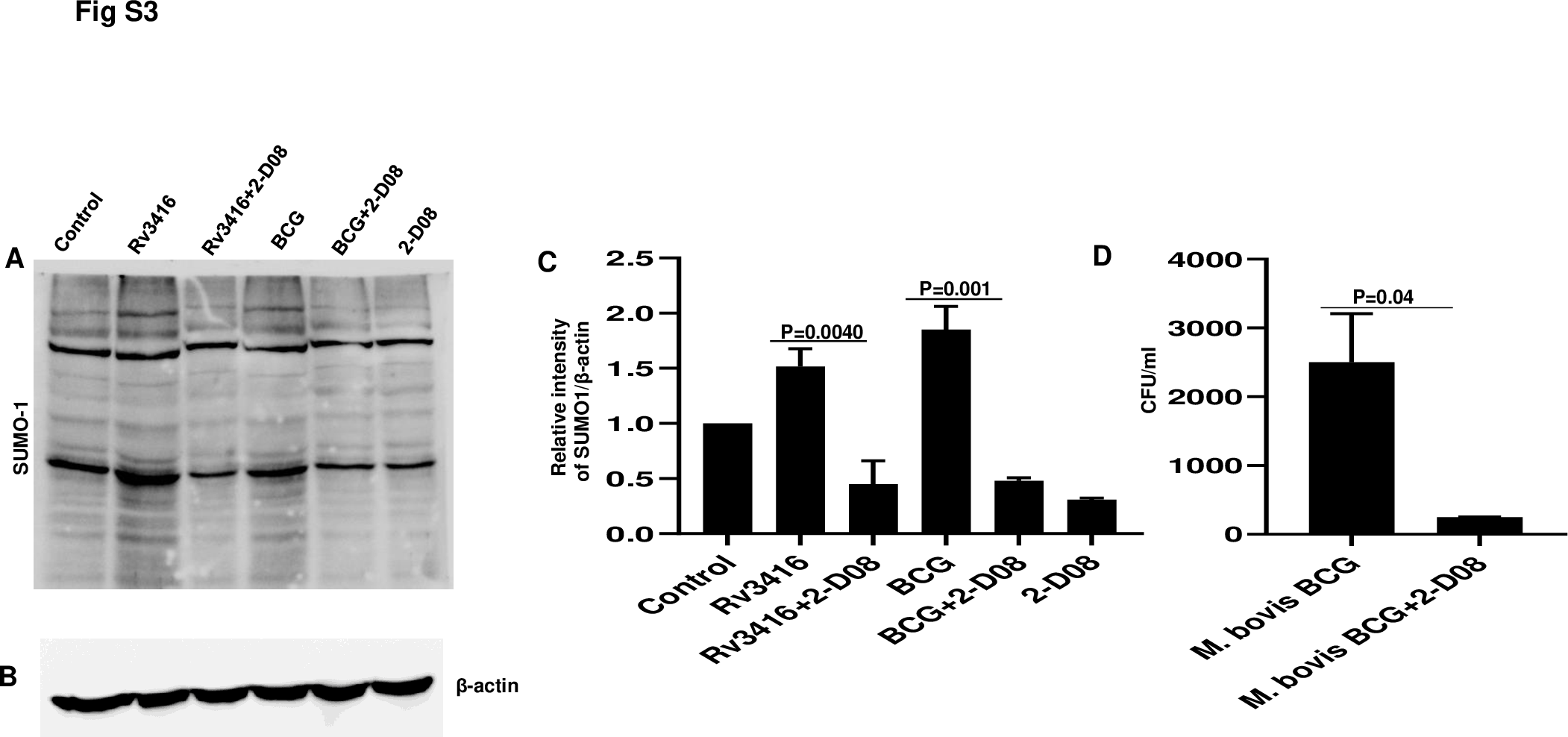

Supplement: S3 Fig — For Panels A THP-1 macrophages were incubated with 25μM 2-D08 for 1h followed by stimulations with 15μg/ml Rv3416 or 2.5MOI BCG for 24h. 30μg total cell extract were western blotted for SUMO1. Data from one of three independent experiments is shown (n = 3). Panel B represents β-actin as loading control. Panels C represent intensities of specific bands plotted as a function of the band intensity of the corresponding loading control. ANOVA with Bonferroni’s post hoc test was performed with 95% confidence interval. For Panel C, P value between groups Rv3416 and Rv3416+2-D08 is P = 0.0040; between groups BCG and BCG+2-D08 is P = 0.001. For Panel D, THP-1 human macrophages were incubated with 25μM 2-D08 for 1h followed by infection with 10MOI BCG for 72h. Serial dilutions of cell lysates were scored for CFU. Data represents mean ± SD of three independent experiments (n = 3). Student’s t test was performed for statistical significance for Panel D. P value between groups BCG and BCG+2-D08 is P = 0.04. (TIF) [file pone.0283448.s003.tif]

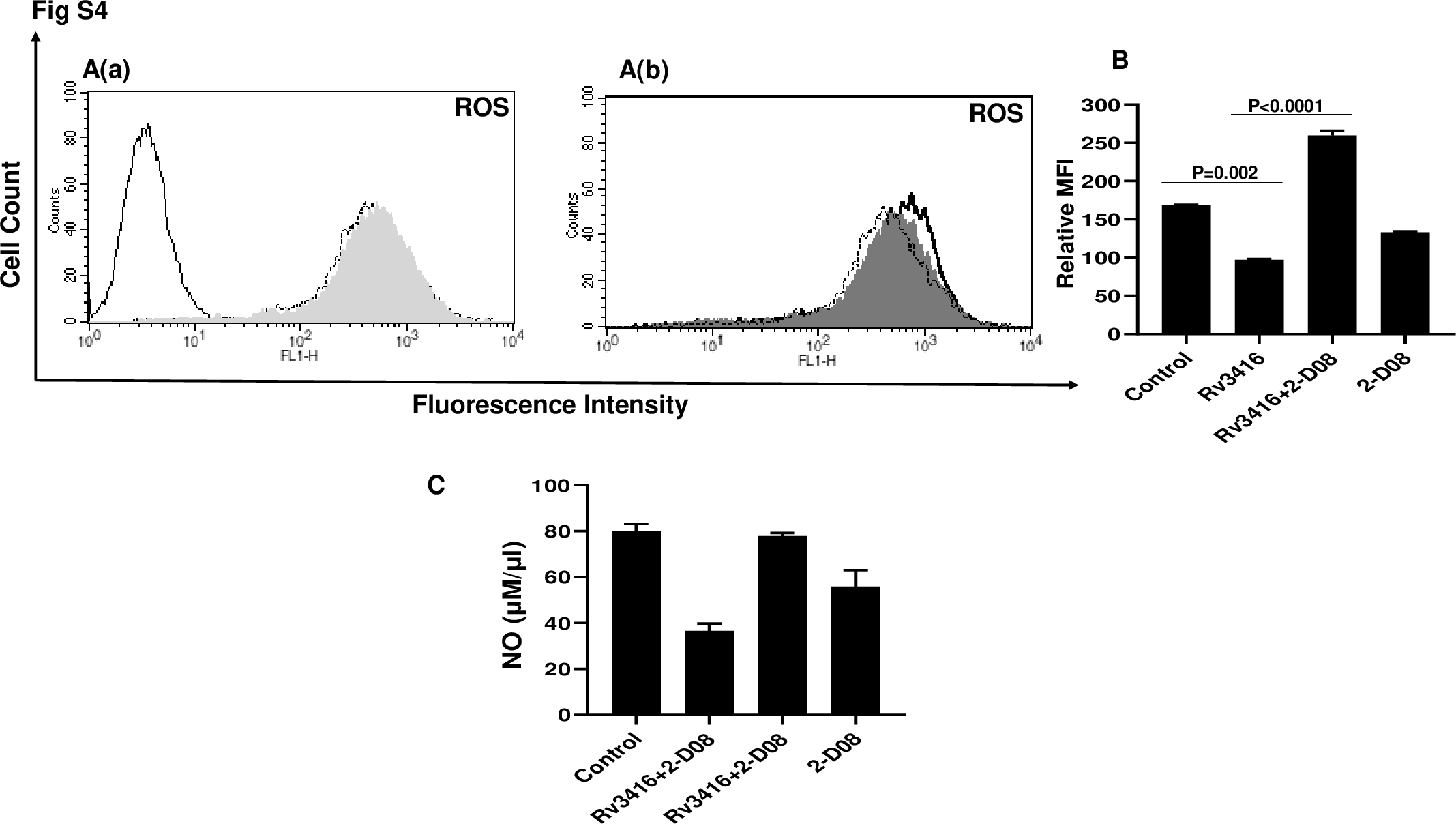

Supplement: S4 Fig — For Panel A, THP-1 human macrophages were incubated with 25μM 2-D08 for 1h followed by stimulation with 15μg/ml Rv3416 for 1h. Oxidative burst was monitored by flow cytometry. In Panel A(a) shaded histogram (light grey) represents unstimulated cells, dotted line represents Rv3416 stimulated cells, while the thin black line depicts unstained cells. In Panel A(b) dark shaded histogram depicts cells treated with 2-D08 only, dotted line depicts Rv3416 treated cells and the thick black line represents Rv3416 stimulated cells pretreated with 2-D08. Multiple measures ANOVA was performed with 95% confidence interval. Bar chart in Panel B represent the Mean Fluorescence Intensities (MFI) of indicated groups as a mean ± SD of three independent experiments (n = 3). For Panel C THP-1 human macrophages were incubated with 25μM 2-D08 for 1h followed by stimulation with 15μg/ml Rv3416 for 24h. Nitric oxide level was monitored by Griess reagent method using spectrophotometer. ANOVA with Bonferroni’s post hoc test was performed was performed with 95% confidence interval. For Panel B, P value between groups Control and Rv3416 is P = 0.002; between group Rv3416 and Rv3416+2-D08 is P<0.0001. (TIF) [file pone.0283448.s004.tif]

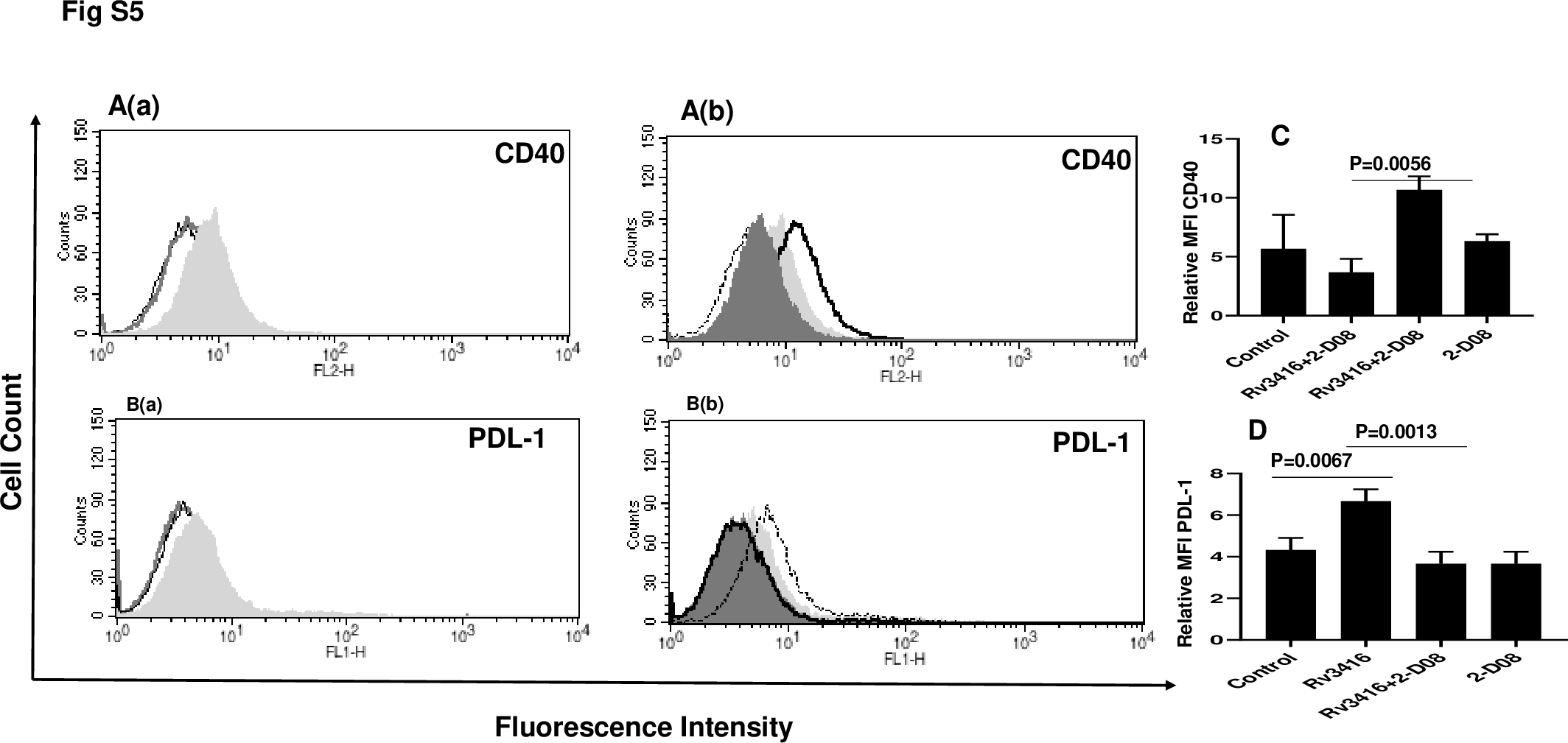

Supplement: S5 Fig — For Panels A and B, THP-1 human macrophages were incubated with 25μM 2-D08 for 1h followed by stimulation with 15μg/ml Rv3416 for 24h. Surface densities of indicated molecules were monitored by flow cytometry. In Panel A(a) light grey shaded histogram depicts unstimulated cells, thick grey line represents Isotype control and thin black line depicts unstained cells. In Panel A(b) light grey shaded histogram depicts unstimulated cells, dotted line represents Rv3416 stimulated cells, thick black line depicts Rv3416 stimulated cells pre-treated with 2-D08 and dark shaded histogram represents cells treated with 2-D08 only. In Panel B(a) shaded histogram depicts unstimulated cells, thick grey line represents Isotype control and thin black line depicts unstained cells. In Panel B(b) light grey shaded histogram depicts unstimulated cells, dotted line represents Rv3416 stimulated cells, thick black line depicts Rv3416 stimulated cells pre-treated with 2-D08 and dark shaded histogram represents cells treated with 2-D08 only. In Panels C and D, bar charts represent Mean Fluorescence Intensities (MFI) of indicated groups as a mean ± SD of three independent experiments (n = 3) for Panels A and B, respectively. ANOVA with Bonferroni’s post hoc test was performed with 95% confidence interval. For Panel C, P value between groups Rv3416 and Rv3416+2-D08 is P = 0.0056. For Panel D, P value between groups Control and Rv3416 is P = 0.0067; P value between groups Rv3416 and Rv3416+2-D08 is P = 0.0013. (TIF) [file pone.0283448.s005.tif]

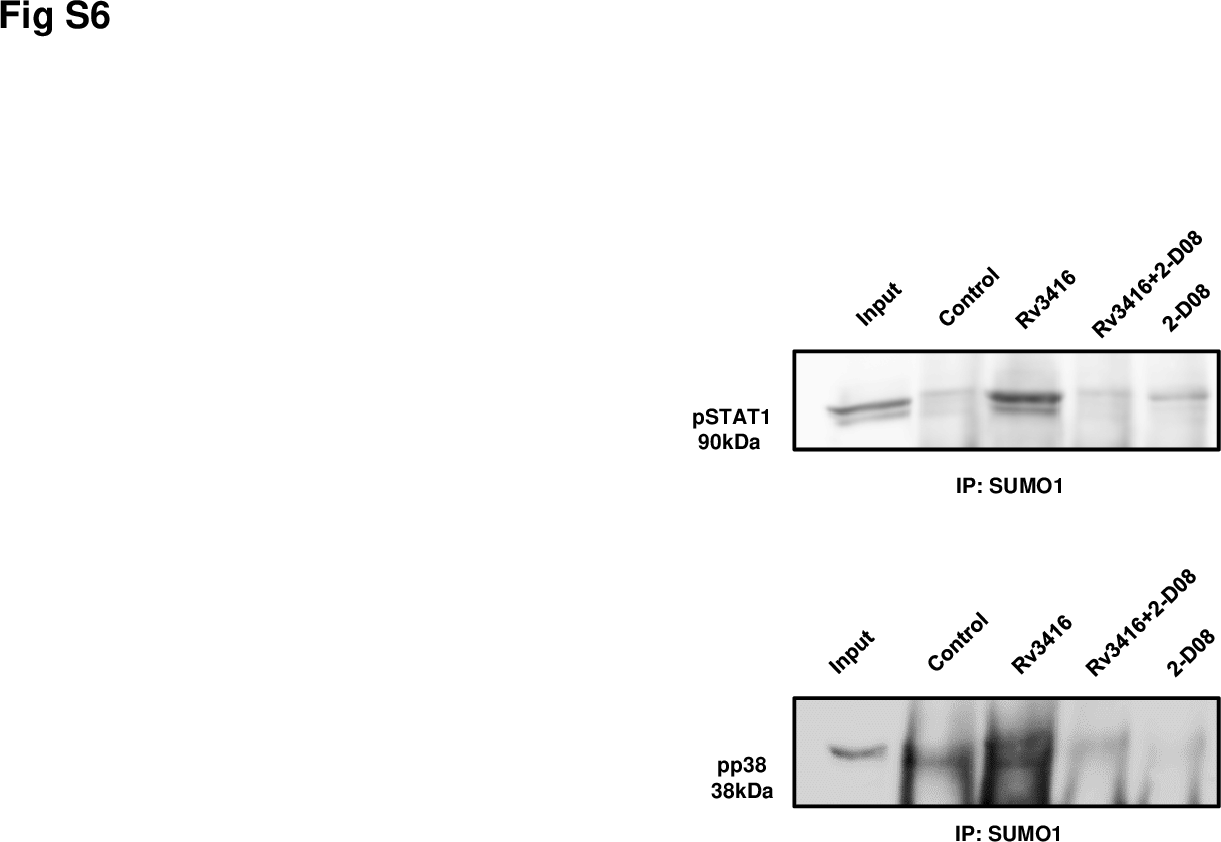

Supplement: S6 Fig — Total cell extract was co-immunoprecipitated with SUMO1 followed by western blotting with pSTAT1 or pp38 antibody. (TIF) [file pone.0283448.s006.tif]

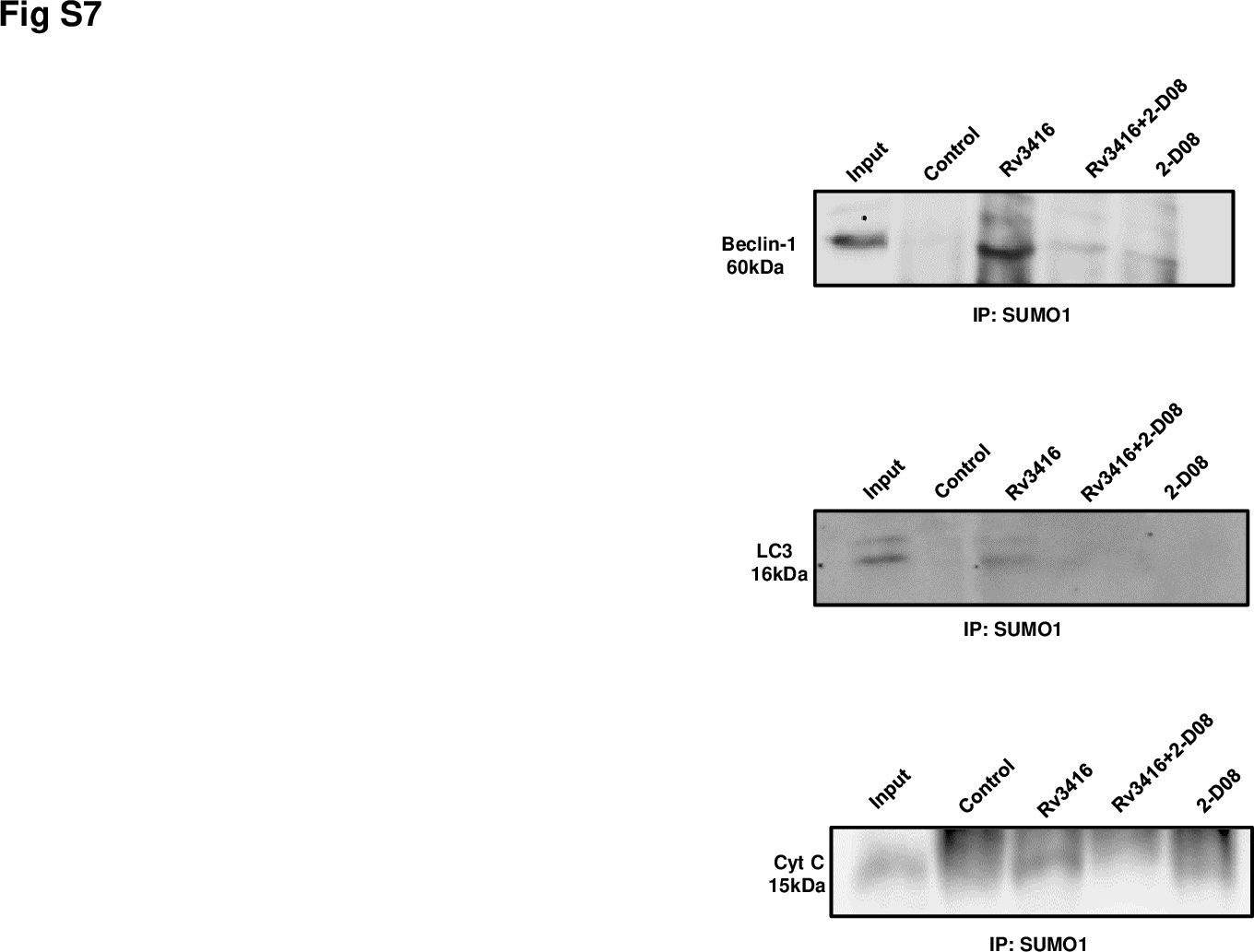

Supplement: S7 Fig — Total cell extract was co-immunoprecipitated with SUMO1 followed by western blotting with Beclin-1, LC3 or Cytochrome C antibody. (TIF) [file pone.0283448.s007.tif]

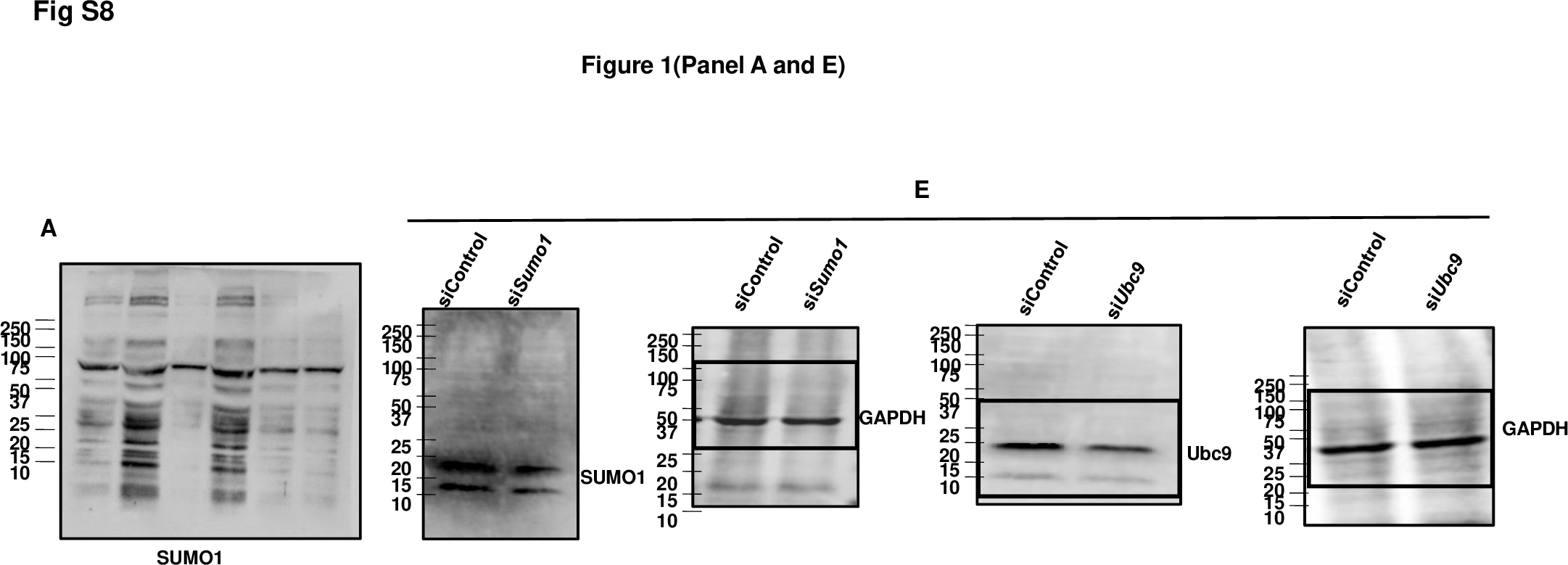

Supplement: S8 Fig — (TIF) [file pone.0283448.s008.tif]

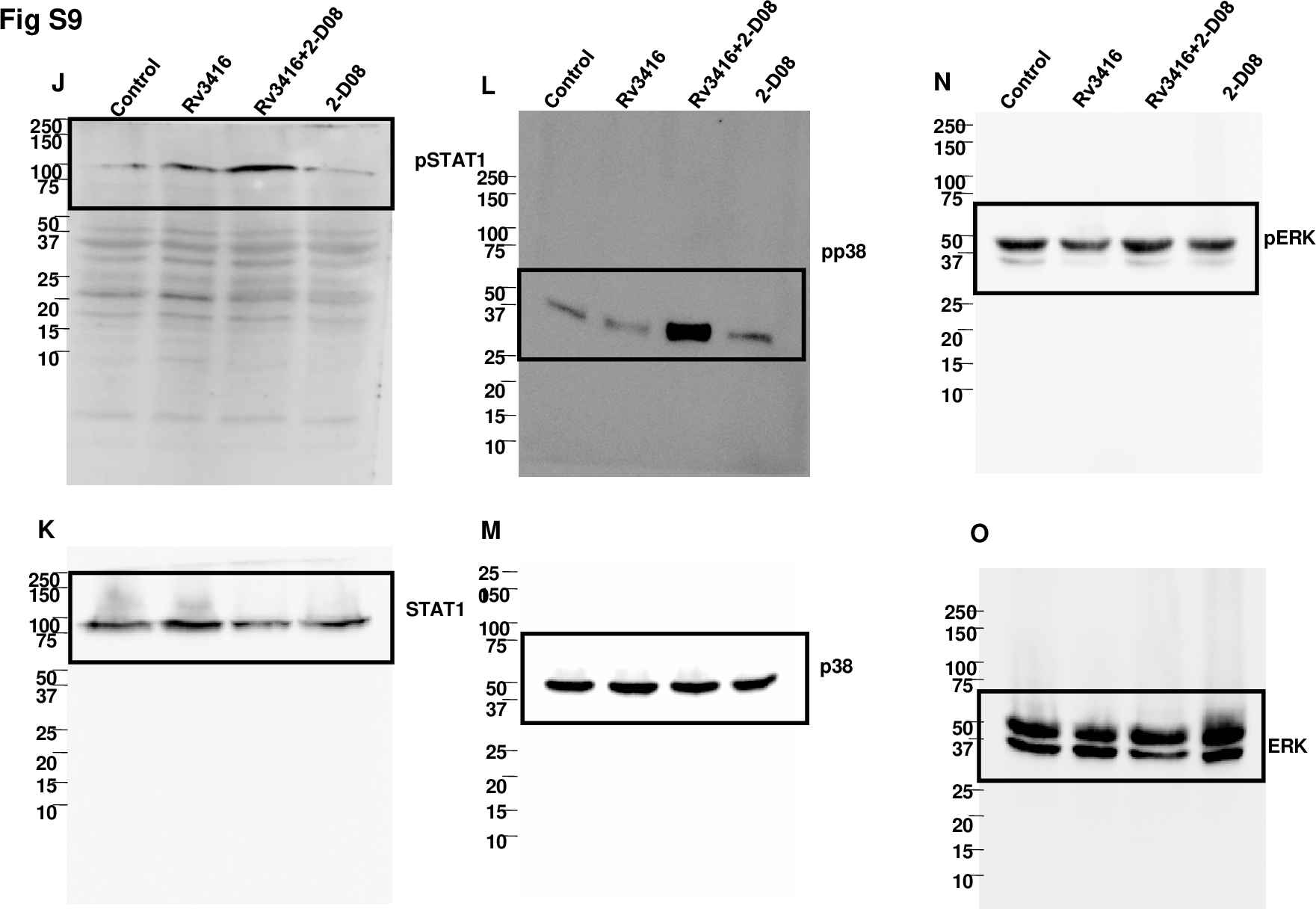

Supplement: S9 Fig — (TIF) [file pone.0283448.s009.tif]

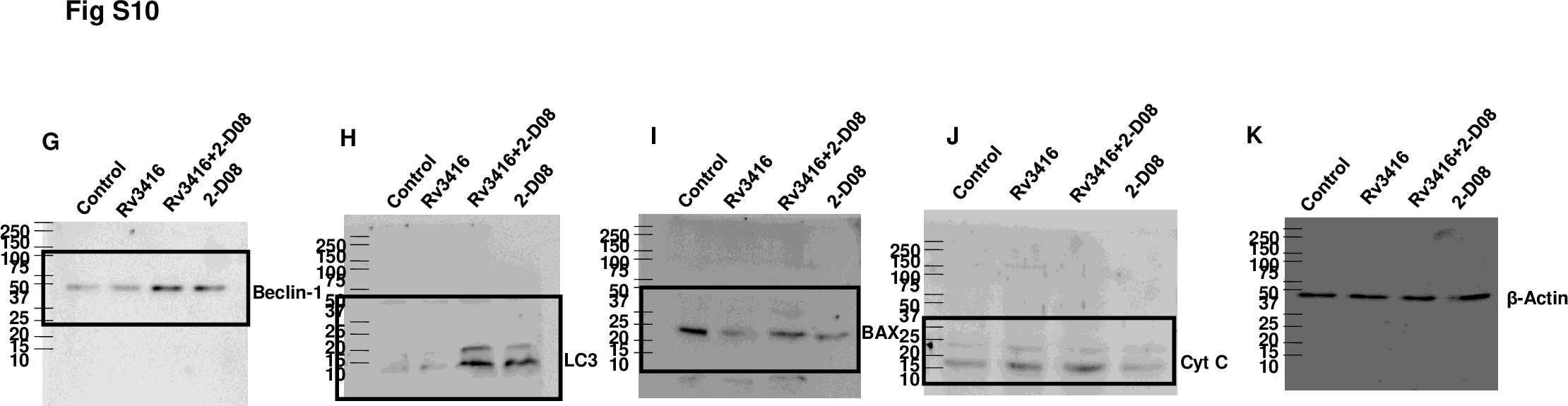

Supplement: S10 Fig — (TIF) [file pone.0283448.s010.tif]

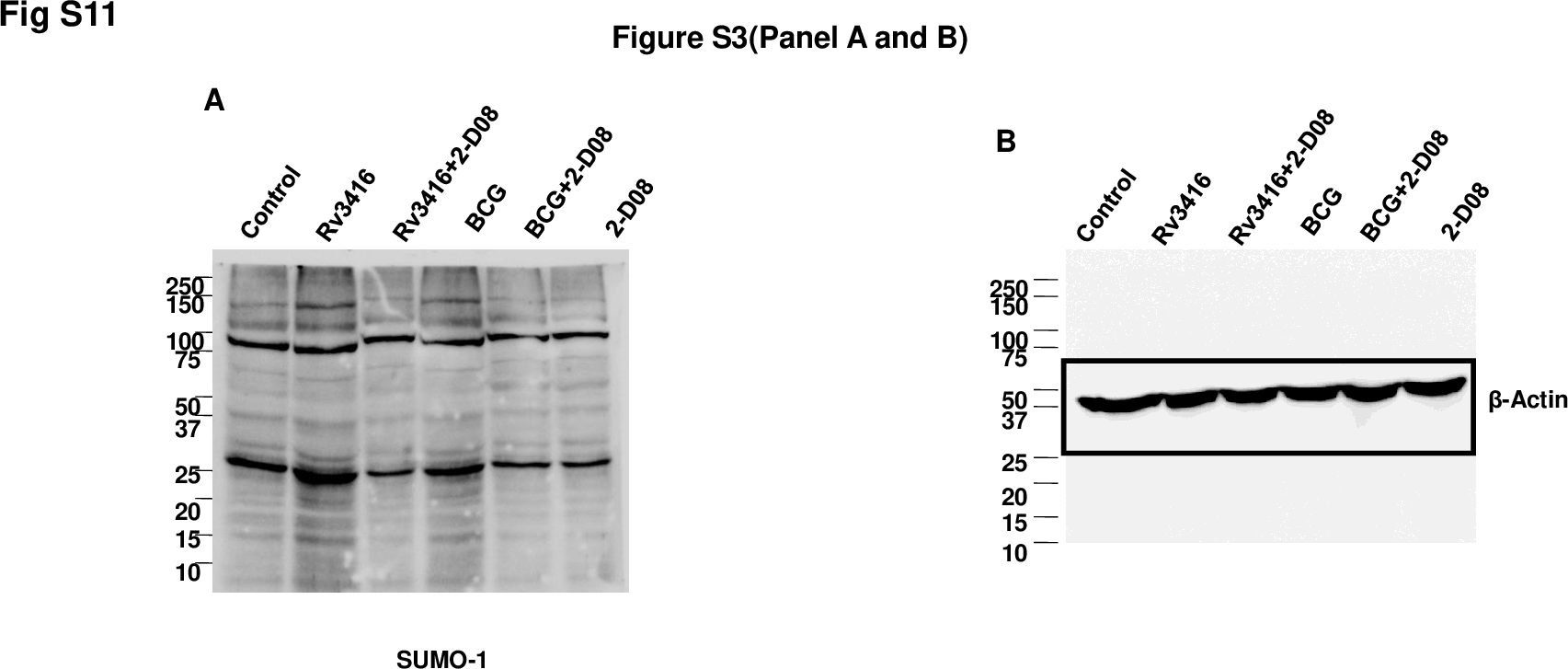

Supplement: S11 Fig — (TIF) [file pone.0283448.s011.tif]
