# Supplementary material for: Mycobacteria modulate SUMOylation to suppresses protective responses in dendritic cells
Source: PLoS One. 2023 Sep 29;18(9):e0283448. doi: 10.1371/journal.pone.0283448 (PMC10540951; doi:10.1371/journal.pone.0283448)
Supplement: S1 File — (PDF) [file pone.0283448.s012.pdf]

Supporting Information

Mycobacteria Modulate SUMOylation to Suppresses Protective Responses in Dendritic Cells

Vandana Anang[1,2,3], Aayushi Singh[2,3], Ankush Kumar Rana[3], Shakuntala Surender Kumar Saraswati[3], Upasana Bandyopadhyay[3], Chaitenya Verma[3], Attinder Chadha[3] and Krishnamurthy Natarajan[1,3]

[3]Infectious Disease Immunology Lab, Dr. B.R. Ambedkar Center for Biomedical Research,

University of Delhi, Delhi 110007, India

[1]To whom correspondence should be addressed: Ms. Vandana Anang, Dr. B. R. Ambedkar Centre for Biomedical Research, University of Delhi, Delhi 110007, India. Email: vandana9293@gmail.comor [1]Prof. Krishnamurthy Natarajan, Dr. B. R. Ambedkar Centre for Biomedical Research, University of Delhi, Delhi 1100
07, India. Tel: +91-11-27666272. Fax: +91-11-27666248. Email: knatarajan@acbr.du.ac.in.
[2]Equal contribution.

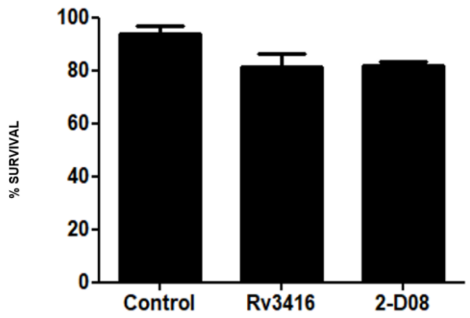

Figure S1: SUMOylation inhibitor 2-D08 does not cause any significant cell death.

BMDCs were incubated with 25µM 2-D08 or Rv3416 for 24h. Cell viability was monitored by MTT assay as described in Materials and Methods.

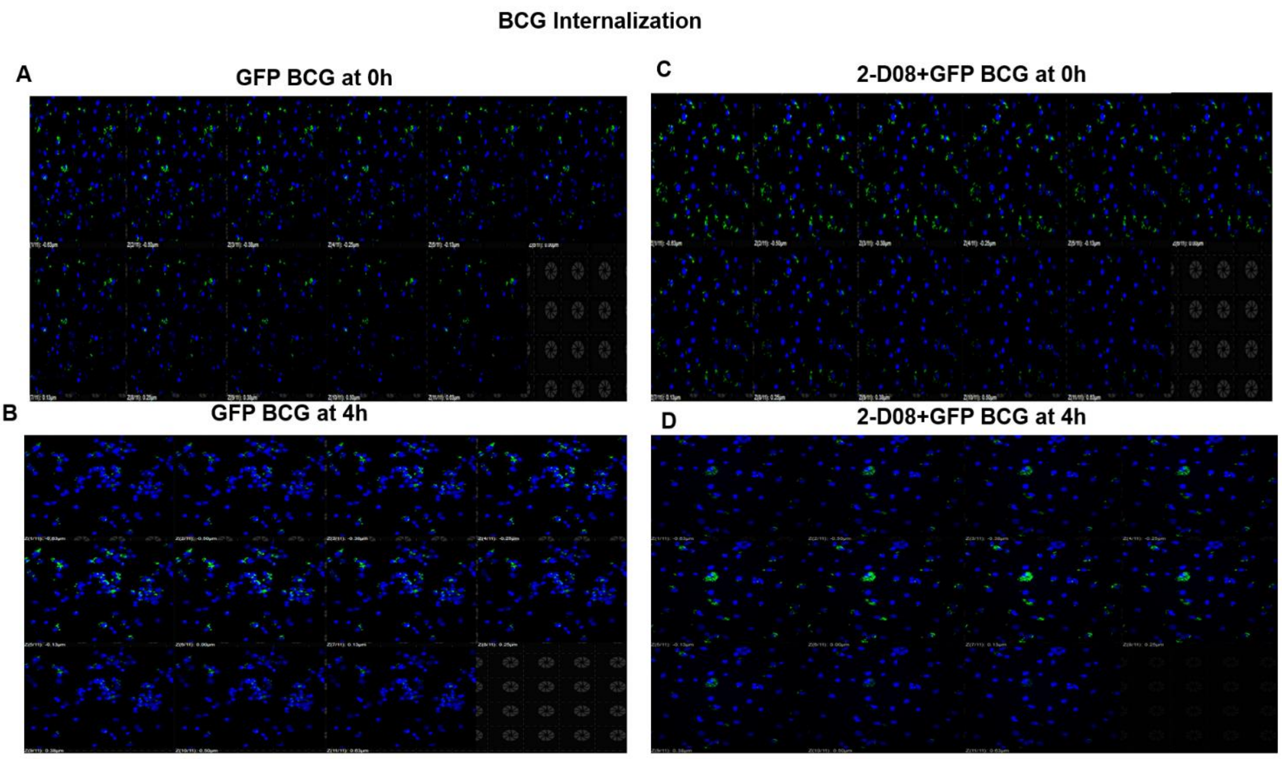

Figure S2: SUMOylation inhibitor 2-D08 does not inhibit internalization of BCG by BMDCs.

For Panel A and B BMDCs were seeded on UV treated coverslips in 12 well culture dishes and infected with 10MOI GFP-BCG for 0h (Panel A) and 4h (Panel B). For Panels C and D, BMDCs were seeded on UV treated coverslips in 12 well culture dishes and incubated with 25µM 2-D08 for 1h followed by infection with 10MOI GFP-BCG for 0h (Panel C) and 4h (Panel D). Internalization of GFP-BCG was monitored using Confocal imaging. Data were analysed using NIS Elements Advanced Research Software. Images show Z-stacks of 1.25µm optical sections. Blue indicates staining of nucleus with DAPI.

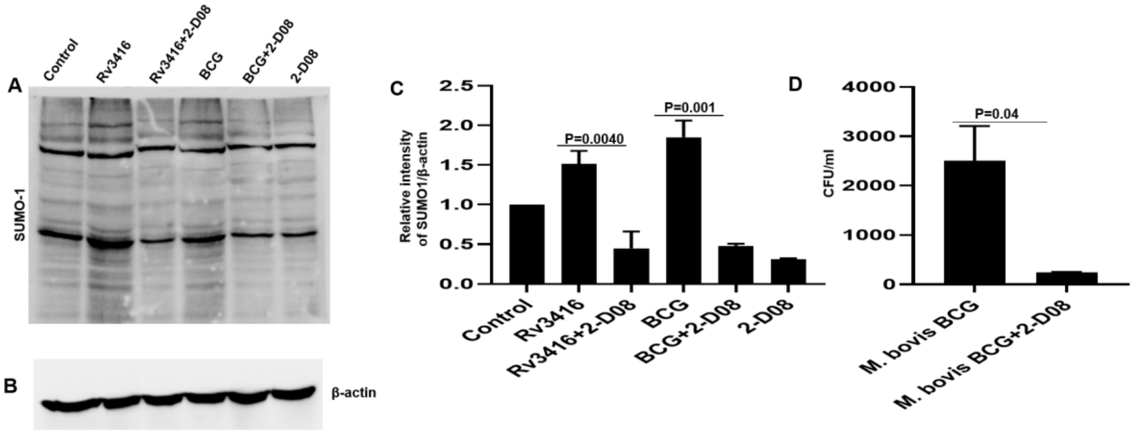

Figure S3: Rv3416 and BCG induces SUMOylation of proteins pathway proteins in THP-1 macrophages and reduces intracellular bacterial survival.

For Panels A THP-1 macrophages were incubated with 25µM 2-D08 for 1h followed by stimulations with 15µg/ml Rv3416 or 2.5MOI BCG for 24h. 30µg total cell extract were western blotted for SUMO1. Data from one of three independent experiments is shown (n=3). Panel B represents β-actin as loading control. Panels C represent intensities of specific bands plotted as a function of the band intensity of the corresponding loading control. ANOVA with Bonferroni's post hoc test was performed with 95% confidence interval. For Panel C, P value between groups Rv3416 and Rv3416+2-D08 is P=0.0040; between groups BCG and BCG+2-D08 is P=0.001. For Panel D, THP-1 human macrophages were incubated with 25µM 2-D08 for 1h followed by infection with 10MOI BCG for 72h. Serial dilutions of cell lysates were scored for CFU. Data represents mean ± SD of three independent experiments (n=3). Student's t test was performed for statistical significance for Panel D. P value between groups BCG and BCG+2-D08 is P=0.04.

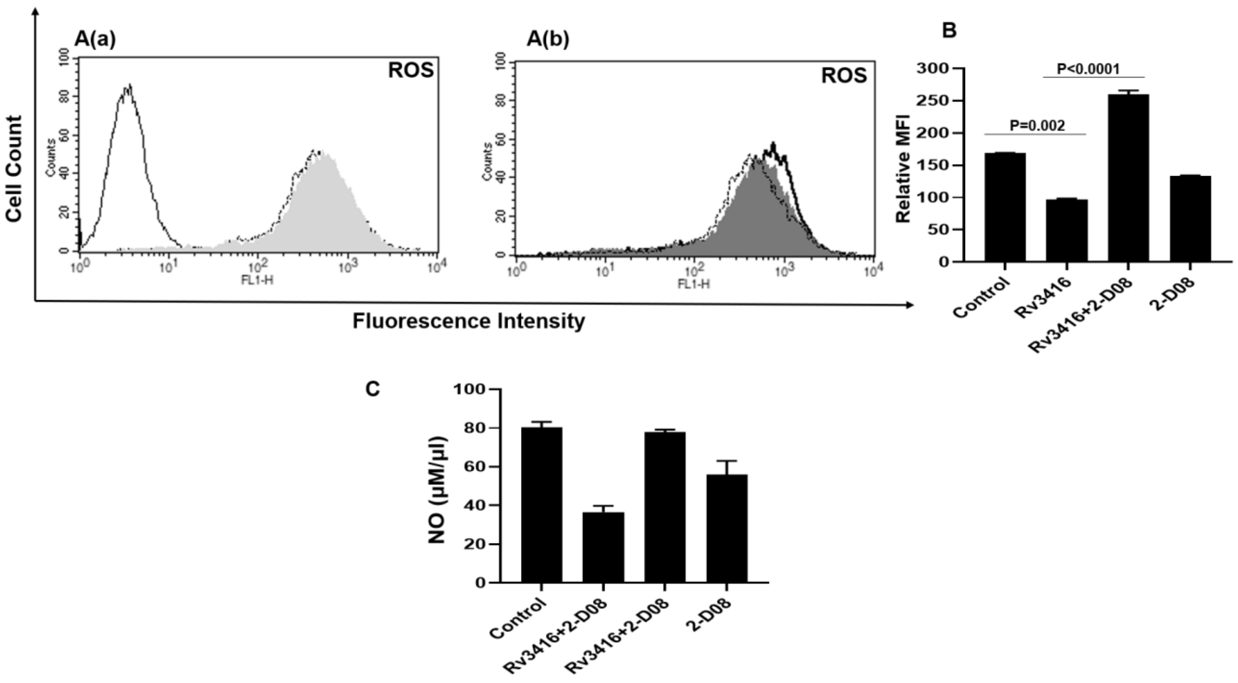

Figure S4: **Inhibiting SUMOylation in Rv3416 stimulated THP-1 human macrophages increases oxidative burst and Nitric Oxide levels.**

For Panel A, THP-1 human macrophages were incubated with 25µM 2-D08 for 1h followed by stimulation with 15µg/ml Rv3416 for 1h. Oxidative burst was monitored by flow cytometry. In Panel A(a) shaded histogram (light grey) represents unstimulated cells, dotted line represents Rv3416 stimulated cells, while the thin black line depicts unstained cells. In Panel A(b) dark shaded histogram depicts cells treated with 2-D08 only, dotted line depicts Rv3416 treated cells and the thick black line represents Rv3416 stimulated cells pretreated with 2-D08. Multiple measures ANOVA was performed with 95% confidence interval. Bar chart in Panel B represent the Mean Fluorescence Intensities (MFI) of indicated groups as a mean ± SD of three independent experiments (n=3). For Panel C THP-1 human macrophages were incubated with 25µM 2-D08 for 1h followed by stimulation with 15µg/ml Rv3416 for 24h. Nitric oxide

level was monitored by Griess reagent method using spectrophotometer. ANOVA with Bonferroni's post hoc test was performed was performed with 95% confidence interval. For Panel B, P value between groups Control and Rv3416 is P=0.002; between group Rv3416 and Rv3416+2-D08 is P<0.0001.

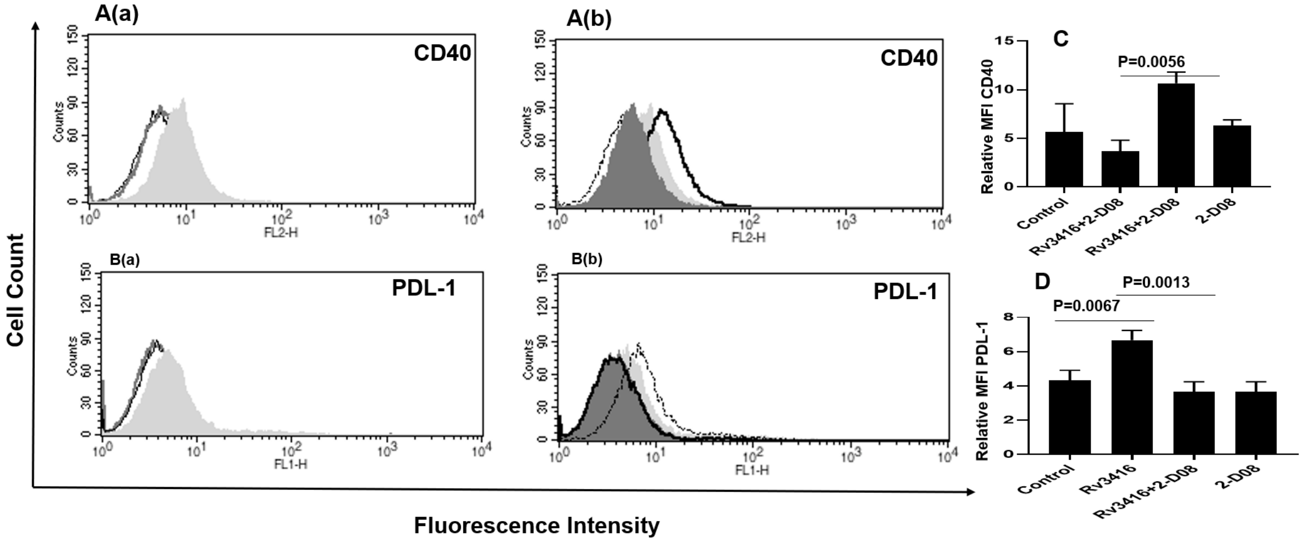

Figure S5. Inhibiting SUMOylation in Rv3416 stimulated THP-1 macrophages modulates the surface expression of co-stimulatory molecules.

For Panels A and B, THP-1 human macrophages were incubated with 25µM 2-D08 for 1h followed by stimulation with 15µg/ml Rv3416 for 24h. Surface densities of indicated molecules were monitored by flow cytometry. In Panel A(a) light grey shaded histogram depicts unstimulated cells, thick grey line represents Isotype control and thin black line depicts unstained cells. In Panel A(b) light grey shaded histogram depicts unstimulated cells, dotted line represents Rv3416 stimulated cells, thick black line depicts Rv3416 stimulated cells pre-treated with 2-D08 and dark shaded histogram represents cells treated with 2-D08 only. In Panel B(a) shaded histogram depicts unstimulated cells, thick grey line represents Isotype control and thin black line depicts unstained cells. In Panel B(b) light grey shaded histogram depicts unstimulated cells, dotted line represents Rv3416 stimulated cells, thick black line

depicts Rv3416 stimulated cells pre-treated with 2-D08 and dark shaded histogram represents cells treated with 2-D08 only. In Panels C and D, bar charts represent Mean Fluorescence Intensities (MFI) of indicated groups as a mean ± SD of three independent experiments (n=3) for Panels A and B, respectively. ANOVA with Bonferroni's post hoc test was performed with 95% confidence interval. For Panel C, P value between groups Rv3416 and Rv3416+2-D08 is P=0.0056. For Panel D, P value between groups Control and Rv3416 is P=0.0067; P value between groups Rv3416 and Rv3416+2-D08 is P=0.0013.

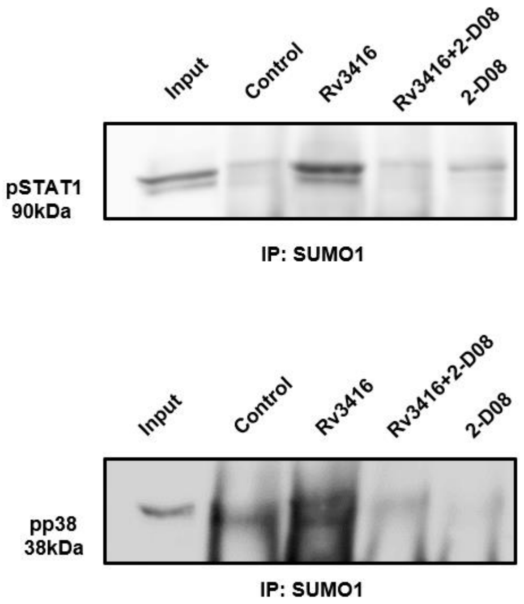

Figure S6. BMDCs were incubated with 25µM 2-D08 for 1h followed by stimulation with 15µg/ml Rv3416 for 1h. Total cell extract was co-immunoprecipitated with SUMO1 followed by western blotting with pSTAT1 or pp38 antibody.

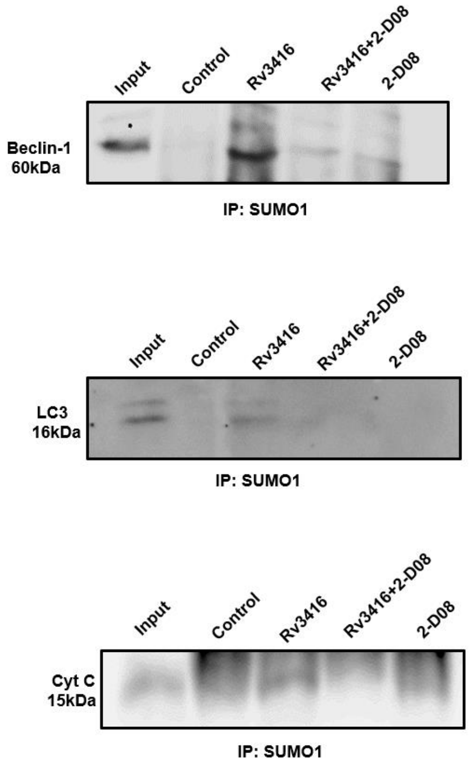

Figure S7. BMDCs were incubated with 25μM 2-D08 for 1h followed by stimulation with 15μg/ml Rv3416 for 1h. Total cell extract was co-immunoprecipitated with SUMO1 followed by western blotting with Beclin-1, LC3 or Cytochrome C antibody.

Figure 1(Panel A and E)

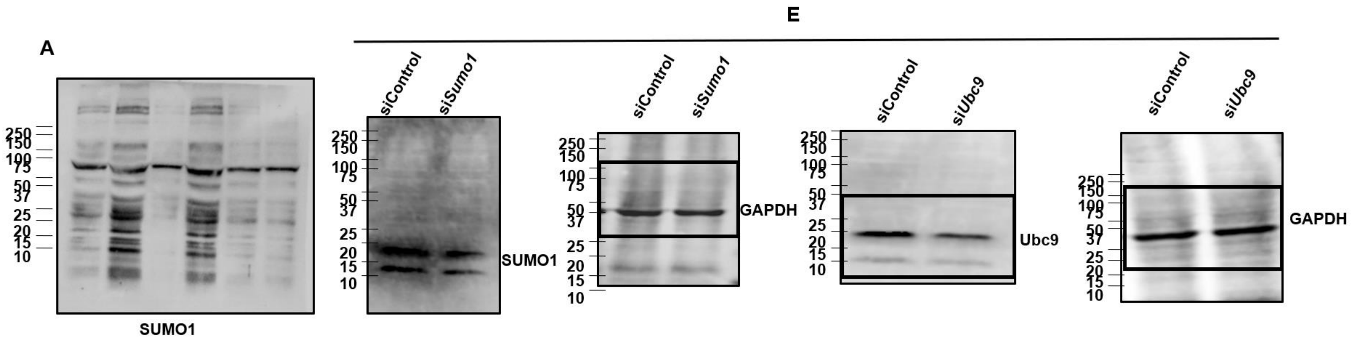

Figure S8: Represents Full size blots for Figure 1 Panels A and E.

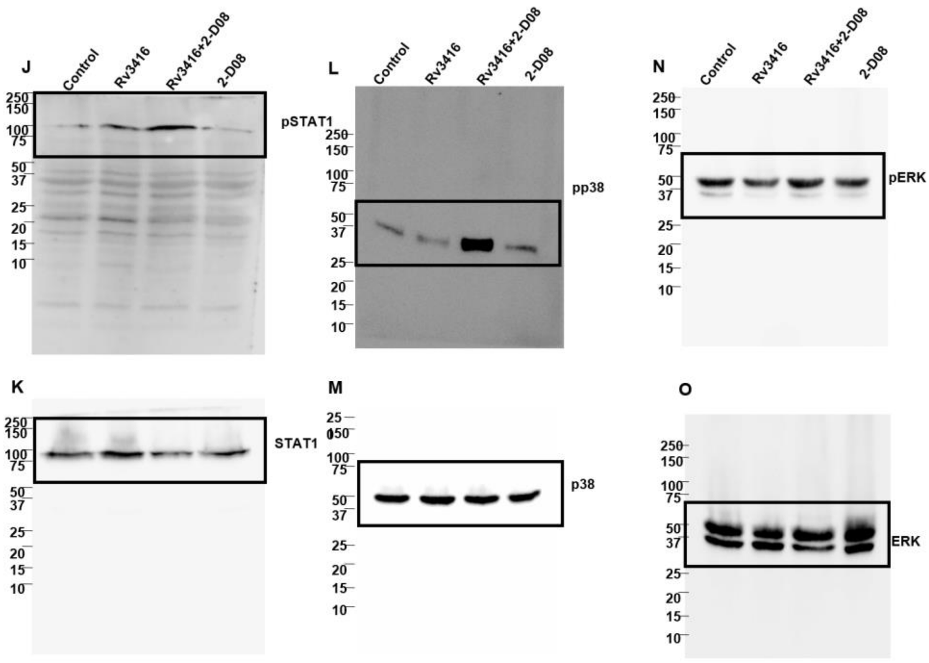

Figure S9: Represents Full size blots for Figure 2 Panel J, Panel K, Panel L, Panel M, Panel N and Panel O, respectively.

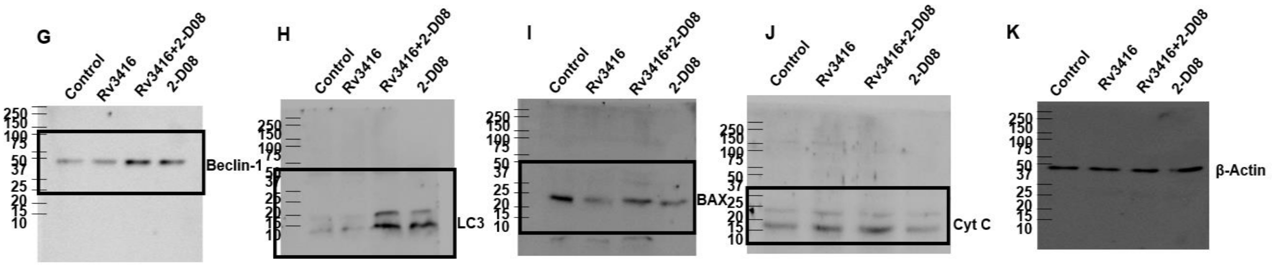

Figure S10: Represents Full size blots for Figure 5 Panel G, Panel H, Panel I, Panel J and Panel K, respectively.

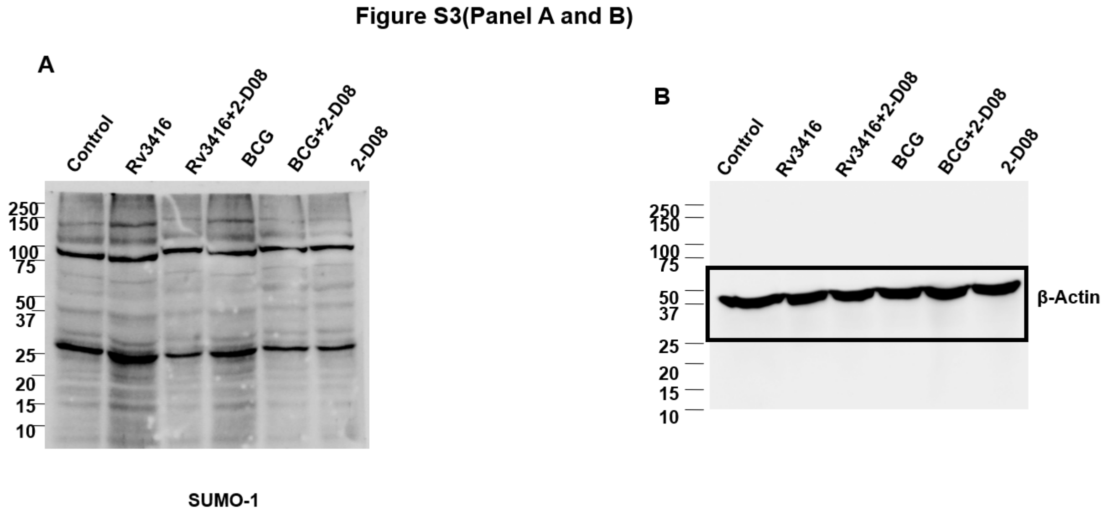

Figure S11: Represents Full size blots for Figure S3 Panel A and B .
